# *Lnc-ITM2C-1* and *GPR55* Are Proviral Host Factors for Hepatitis C Virus

**DOI:** 10.3390/v11060549

**Published:** 2019-06-13

**Authors:** Pan Hu, Jochen Wilhelm, Gesche K. Gerresheim, Lyudmila A. Shalamova, Michael Niepmann

**Affiliations:** 1 Institute of Biochemistry, Medical Faculty, Justus-Liebig-University, Friedrichstrasse 24, 35392 Giessen, Germany; gesche.gerresheim@gmx.de (G.K.G.); ludmilashalamova@gmail.com (L.A.S.); 2 Universities of Giessen and Marburg Lung Center (UGMLC), German Center for Lung Research (DZL), 35392 Giessen, Germany; jochen.wilhelm@patho.med.uni-giessen.de

**Keywords:** HCV, replication, lncRNA, *LOC151484*, innate immunity, *GPR55*, cannabinoid receptor

## Abstract

Multiple host factors are known to play important roles in hepatitis C virus (HCV) replication, in immune responses induced by HCV infection, or in processes that facilitate virus escape from immune clearance, while yet only few studies examined the contribution of long non-coding RNAs (lncRNAs/lncRs). Using microarrays, we identified lncRNAs with altered expression levels in HCV replicating Huh-7.5 hepatoma cells. Of these, lncR 8(*Lnc-ITM2C-1*/*LOC151484*) was confirmed by quantitative real-time PCR (qRT-PCR) to be upregulated early after HCV infection. After suppressing the expression of lncR 8, HCV RNA and protein were downregulated, confirming a positive correlation between lncR 8 expression and HCV replication. lncR 8 knockdown in Huh-7.5 cells reduced expression of the neighboring gene G protein-coupled receptor 55 (*GPR55*) mRNA level at early times, and leads to increased levels of several Interferon stimulated genes (ISGs) including *ISG15*, *Mx1* and *IFITM1*. Importantly, the effect of lncR 8 on ISGs and *GPR55* precedes its effect on HCV replication. Furthermore, knockdown of *GPR55* mRNA induces ISG expression, providing a possible link between lncR 8 and ISGs. We conclude that HCV induces lncR 8 expression, while lncR 8 indirectly favors HCV replication by stimulating expression of its neighboring gene *GPR55*, which in turn downregulates expression of ISGs. The latter fact is also consistent with an anti-inflammatory role of *GPR55*. These events may contribute to the failure to eliminate ongoing HCV infection.

## 1. Introduction

First identified in 1989, Hepatitis C virus (HCV) is an enveloped virus belonging to the *Flaviviridae* family [1,2]. The components of the HCV virion particle include the 9.6 kb single-stranded HCV RNA genome of positive polarity and some HCV non-structural proteins [3,4]. The HCV RNA moves to ribosomes after viral entry, and serves as a messenger RNA (mRNA) for translation of the viral proteins [3,5,6]. The encoded polyprotein is processed by viral and host proteases into 10 mature proteins, core, E1, E2, p7, NS2, NS3, NS4A, NS4B, NS5A, and NS5B [7,8,9]. Negative strand RNA intermediates are generated which then act as templates for the synthesis of new positive strand genomic RNA at the endoplasmic reticulum(ER)-derived membranous webs [3,7]. Viral assembly and release are the last steps of a complete HCV viral life cycle [3,5,6,7]. 

During its life cycle, the cell develops several mechanisms to recognize the virus and fight against it. The tightly coordinated innate immune signaling pathways in the liver provide the first and significant line of host defense against HCV [10,11], while the adaptive immune response emerges over several weeks [12]. Upon HCV infection, specific pathogen-associated molecular patterns (PAMPs) of HCV can be sensed by different pattern recognition receptors (PRRs), like retinoic acid-inducible gene I (*RIG-I*), melanoma differentiation factor 5 (*MDA5*), and toll-like receptor 3 (*TLR3*), leading to the production of pro-inflammatory cytokines, chemokines, and interferon (IFN), which include Type I IFN (*IFNα*, *IFNβ*, and others), Type II IFN (*IFNγ*), and Type III IFN (*IFNλ*) [12,13,14,15]. After triggering the JAK-STAT signaling pathway, the final outcome of the IFN signaling is the induction of hundreds of IFN-stimulated genes (ISGs), which serve as direct effectors of the IFN antiviral defense [10,12,14,15]. Antiviral ISGs may target many steps in the HCV life cycle to limit viral replication or promote the IFN antiviral ability [13,14,16]. IFN signaling and the subsequent expression of ISGs are central in this antiviral defense [17]. Only combined ISGs can induce a strong antiviral response, while the effect of a single ISG is weak [14,18].

In spite of activated immune response, 70–80% of infected patients develop chronic infection without clearance of HCV, including chronic hepatitis, cirrhosis and hepatocellular carcinoma (HCC). HCV remains a global health issue affecting approximately 2% of the global population [1,14,19]. The co-existence of high viral loads and high ISG expression reflects the failure of the innate immune response in clearing HCV [10], suggesting strategies used by HCV to evade the host immune response [11]. It was shown that the ineffectiveness of the innate immune response can be achieved by cleavage of MAVS by NS3/4A protease, by an ISG translation block mediated by the noncanonical cellular sensors dsRNA-activated protein kinase R (*PKR*) and DEAD box RNA helicase 3 (*DDX3X*), or by ISGs like ubiquitin specific peptidase 18 (*USP18*) that downregulates the IFN pathway response as a negative feedback to ensure homeostasis of the cellular immune response [4,11,13,14,16,20]. Autophagy induced by HCV might also be involved in the suppression of type I IFN production [18]. Moreover, HCV related exosomes also contribute to the immune escape [20]. 

Constituting about 65% of the human transcriptome, long non-coding RNA (lncRNA) is defined as RNA with more than 200 nucleotides in length and lacking protein coding capacity or only containing small open reading frames (ORFs) [4]. LncRNAs can regulate chromatin remodeling, transcription in *cis* or *trans*, translation, or serve as enzyme cofactors [21]. Increasing evidence suggests that cellular lncRNAs may be deregulated in response to viral replication or to the antiviral pathways induced by infection [4,13]. They may function in the HCV life cycle, the antiviral immune response induced by HCV, or in HCV immune escape, finally exerting a proviral or antiviral role [4,14]. LncRNAs, like growth arrest-specific 5 (*GAS5*), BST2 interferon stimulated positive regulator (*BISPR*), lncRNA#32/*LUARIS*, and *lncITPRIP-1* can suppress HCV replication by different mechanisms. *GAS5* binds HCV NS3 protein to inhibit its functions or binds *miR-222* to release p27 protein, *lncITPRIP-1* enhances the innate immune response by *MDA5* oligomerization and activation [22,23,24]. LncRNA#32/*LUARIS* controls the expression of several ISGs [25], while *BISPR* appears to increase the expression of a single target gene, ISG *BST2*, and thereby leads to decreased virion release [13]. In fact, GAS5 was also reported to positively regulate IFN responses in esophageal squamous cell carcinoma [26]. In contrast, negative regulator of interferon response (*NRIR*) (also known as *lncRNA-CMPK2*) and eosinophil granule ontogeny transcript (*EGOT*) are proviral lncRNAs which negatively regulate ISGs and thus antagonize the antiviral response [4,14,15]. *NORAD* binds *miR-373*, resulting in release of their common target *Wee1* and thereby deregulation of cell growth in HCV infected cells [27]. Interestingly, *BISPR* and *NRIR* are also bona-fide ISGs themselves [14]. In fact, investigation in esophageal squamous cell carcinoma also supports that *GAS5* is an ISG which regulates the expression of other ISGs [26]. Taken together, accumulating data suggest a critical role of lncRNAs during HCV infection. However, only a small number of lncRNAs, even less for HCV-related lncRNAs, has been functionally studied [14]. 

In this study, we examined cellular lncRNAs with altered expression after fully established infection with HCV to identify additional lncRNAs that may regulate different steps of the HCV life cycle and the innate immune response. Two novel lncRNA candidates identified in this study, with anti- or proviral function for HCV replication, underline an involvement of lncRNAs in the battle of HCV and host cells.

## 2. Materials and Methods

### 2.1. Cell Culture

Human hepatocarcinoma derived Huh-7.5 cells and naïve Huh-7 cells, kindly provided by Charles Rice (Rockefeller University, New York, USA)and Ralf Bartenschlager (Heidelberg, Germany) respectively, were maintained in Dulbecco’s modified Eagle’s medium (DMEM) (Life Technology) supplemented with 10% fetal bovine serum (FBS) and 1% penicillin–streptavidin (10,000 U/mL), and grown at 37 °C in a 5% CO_2_ incubator. 

### 2.2. Plasmid and In Vitro Transcription

Plasmid pFK-JFH1-J6 C-846_dg (briefly: Jc1) as previously described [28], kindly provided by Ralf Bartenschlager (Heidelberg, Germany), was used to generate full-length HCV Jc1 genomes(J6/JFH1 chimeric genotype 2a) by in vitro transcription. 

The Jc1 plasmid was first digested with Mlu I-HF (NEB) for 2 hours (hrs) at 37 °C. Linearized DNA was purified by phenol/chloroform extraction and ethanol precipitation. Then, the concentration of dissolved DNA was measured by Qubit 2.0 Fluorimeter (ThermoFisher). The DNA size and linearization were checked on agarose gels. 

In vitro transcription was performed using T7 RNA Polymerase (ThermoFisher) in the presence of 3.75 mM of each NTP, additional 5 mM MgCl_2_ and 10 mM DTT, and 30 ng/µL of linearized plasmid DNA. After 2 h of incubation at 37 °C, another 1 U/µL of T7 RNA Polymerase was added for 2 h more. Template DNA was then digested by 2 U RNase-free DNase I (NEB) per 1 µg of DNA for 1 h at 37 °C. HCV full-length Jc1 RNA transcripts were dissolved in equal amounts of RNase-free water. After removing the enzymes using GeneJET RNA Clean-up Kit (ThermoFisher), transcripts were checked for integrity by agarose gel electrophoresis and quantified by Qubit Fluorimeter.

### 2.3. Infectious HCV in Cell Culture

The Jc1 in vitro-transcribed RNA was transfected into Huh-7.5 cells by electroporation. The culture supernatants collected at 6 day (d) after transfection were distributed into split Huh-7.5 cells. After additional multiplication passages on naïve cells, the cell-free supernatants containing HCV were concentrated approximately 50-fold using Amicon Ultra-15 Centrifugal Filters (Millipore, Billerica, MA, USA). Aliquots were stored at −80 °C until use. Virus titers were determined by focus-forming units (FFU) assay. Huh-7.5 cells were seeded at 0.25 × 10^5^ cells per well in 24-well plates and cultured overnight. Test samples were diluted serially 10-fold and each dilution was inoculated into the cells. After incubation for 4 h at 37 °C, the cells were supplemented with fresh complete DMEM and cultured for 48 h. The cells were then immunofluorescence-stained for HCV NS5A. HCV-positive foci were manually counted under a fluorescence microscope. The virus titer was expressed as focus-forming units per milliliter of supernatant (FFU/mL), as determined by the average number of NS5A-positive foci detected in a whole well.

### 2.4. Oligonucleotides (Oligos)

miR-122 RNA oligos were supplied by biomers.net (Germany). The sequences were: miR-122 mat, 5′-(phos) UGGAGUGUGACAAUGGUGUUUG-3′, miR-122*, 5′-(phos) AACGCCAUUAUCACACUAAAUA-3′. Duplexes were formed by annealing same amounts of the guide (mat) and its complementary passenger strand (*) in a thermocycler by a steady temperature decrease from 90 °C to 4 °C (1 °C per minute). 

The Locked nucleic acid (LNA) mixmer oligo for sequestering miR-122 was ordered from Exiqon (Denmark). The sequence was:

5′-+C*C*A*+T*T*G*+T*C*A*+C*A*C*+T*C*+C-3′, where (+) indicates a following LNA residue and G*, A*, T*, C* indicate phosphorothioate DNA bases. 

LNA^TM^ long RNA GapmeR (GmR) oligos targeting different lncRNA candidates were designed using online Antisense GmR Designer (https://www.qiagen.com/de/shop/genes-and-pathways/custom-products/custom-assay-products/antisensegapmerdesigner/) and purchased from Qiagen (Germany). The sequences of the GmRs were:

GmR Negative Control A (Neg. ctr. GmR): 5′-AACACGTCTATACGC-3′;
GmR 1 for lncR 3/*LINC00222* (lncR 3-GmR 1): 5′-GCGTGATTAAATGGAT-3′;
GmR 2 for lncR 3/*LINC00222* (lncR 3-GmR 2): 5′-GACGATAAGAGGTAAC-3′;
GmR 1 for lncR 7/*Lnc-SLC12A7-4* (lncR 7-GmR 1): 5′-TGATTAACAGAACGGA-3′;
GmR 2 for lncR 7/*Lnc-SLC12A7-4* (lncR 7-GmR 2): 5′-ATAAGTGTCTAGTTAG-3′;
GmR 1 for lncR 8/*Lnc-ITM2C-1*(lncR 8-GmR 1): 5′-GTTACCAGTGAAGCGG-3′;
GmR 2 for lncR 8/*Lnc-ITM2C-1* (lncR 8-GmR 2): 5′-TCGGATTGGTCACATG-3′;
GmR 1 for lncR 10/*ZNF252P-AS1* (lncR 10-GmR 1): 5′-GTTAATCTGATCTTGC-3′;
GmR 2 for lncR 10/*ZNF252P-AS1* (lncR 10-GmR 2): 5′-TCTGAGCTTGATCACT-3′;
GmR 1 for *GPR55* (GPR55-GmR 1): 5′-GGCGAATCAGATTAAT-3′;
GmR 2 for *GPR55* (GPR55-GmR 2): 5′-AGGACCATCTTGAATG-3′;

Primers were purchased from biomers.net. Primers used for reverse-transcription (RT) reaction and qRT-PCR of lncRNA candidates are listed in Table 1 (Primers for lncRs 1, 4, 5, 6, and 9 with failed amplification are not shown). Primers for other genes are listed in Table 2. Most primers were designed by the PrimerPremier5 program (United Kingdom). Primers to amplify *GAS5*, small nucleolar RNA U99, H/ACA box 57 (snoRNA U99, *U99*), ISG15 ubiquitin-like modifier (*ISG15*), MX dynamin like GTPase 1 (*Mx1*), and interferon induced transmembrane protein 1 (*IFITM1*) fragments were obtained from previous reports [15,22,29,30]. Primers for Integral membrane protein 2C (*ITM2C*), G protein-coupled receptor 55 (*GPR55*), C-X-C motif chemokine ligand 10 (*CXCL10*), melanoma differentiation-associated protein 5 (*MDA5*, also named *IFIH1*), interferon beta 1, fibroblast (*IFN-β*) were from PrimerBank (https://pga.mgh.harvard.edu/primerbank/). Two different sets of primers targeting two sites of the sequences were designed for lncRs 3, 7, 8, and 10. qRT-PCR detecting expression after miR-122 with or without HCV treatment, and detecting cytoplasm/nucleus location were performed using 5′ side primers. To test the effect after GmR knockdown, 5′ side primers for lncRs 3 and 7, 3′ side primers for lncRs 8 and 10 were used.

### 2.5. Cell Treatment

To identify HCV altered transcriptome, transfection of 500 ng miR-122 duplex into Huh-7.5 cells in T175 flask was performed using Lipofectamine 2000 (Invitrogen) 24 h prior to HCV RNA electroporation. Cells were transfected at about 70% confluency. Oligos and Lipofectamine were first prepared as master mixtures in separate tubes in serum/antibiotic-free DMEM (50 μL/reaction). After 5 minutes (min) at room temperature, each sample was mixed together with Lipofectamine and incubated for 15 min. Then, 100 μL Lipofectamine-oligo mixed solution was carefully applied to the cells dropwise. At 3 h post transfection, the cells were washed in phosphate buffered saline (PBS), and fresh medium was added. The in vitro transcribed Jc1 HCV RNA together with miR-122 duplex, or miR-122 duplex only, were transfected into 400 µL of cells at 1.0 × 10^7^ cells/ml by electroporation one day later. miR-122 duplexes with or without 8 µg HCV RNA were separately prepared for each treatment. Anti-miR-122 LNA mixmer, which sequesters endogenous miR-122 and by that disables HCV replication, was also used to treat cells alone or with HCV transfection. The setting for Gene Pulser Xcell (Biorad, USA) was: square wave, 270 V, 20 ms, 1 Pulse, 4 mm cuvette. The cells were washed with PBS to remove dead cells at 6 h post incubation (hpi). Cells were further incubated with complete DMEM for 72 h, and another round of miR-122 duplex transfection was carried out to compensate for miR-122 loss and degradation after three days incubation in cells. HCV infection was allowed to proceed and cells were harvest 48 h later (i.e., 6 d after HCV RNA transfection). 

For knockdown experiments, cells were seeded at 1.5 × 10^5^ cells/mL in 12-well plates 24 h before GmR treatment. 50 pmol of GmRs targeting lncRNA candidates in a final volume of 1 mL were transfected with Lipofectamine 2000 24 h prior to HCV transfection or infection. The medium was not supplemented with antibiotics. Medium from the cells was then substituted by fresh DMEM supplemented with antibiotics and FBS, and full-length HCV genome was transfected at 0.375 µg/well using Lipofectamine 2000. Cells were harvested after 12, 24, and 48 h incubation. 

To study HCV infection, cells were infected with HCV at the multiplicity of infection (moi) of 0.3 for 4 h. After 4 h of infection, medium supernatants were removed and fresh medium was added to the cells. Cell supernatants and pellets were harvested at the indicated times post-infection. A replication defective mutant version of the HCV genome (NS5B replicase inactivating "GND" mutation) was also prepared to infect cells. Pathogen associated molecular pattern (PAMP) poly (I:C) (Invivogen) was also used to treat Huh-7.5 cells and Huh-7 cells at 2.5 µg or 5 µg per well for 8 h. In experiments with Janus kinase/signal transducers and activators of transcription (JAK–STAT) inhibitor, Huh-7.5 cells were treated with the JAK inhibitor ruxolitinib (Invivogen) (0.8 µM) for 1 h, with a subsequent treatment with IFN-α2 (100 units/mL) or mock control for 8 h followed by harvest of RNA.

### 2.6. RNA Samples, DNA Removal, and cDNA Preparation

Total RNA was isolated from cells using TRIzol (Invitrogen). After DNase I treatment, the total RNA was purified using GeneJET RNA Clean-up Kit. Nuclear and cytoplasmic cell fractionation was obtained using the Paris kit following the manufacturer’s instructions (Life Technologies). RNA integrity was checked by agarose gel electrophoresis, and RNA concentrations were measured by Qubit 2.0. Reverse transcription (RT) was performed using the qScript Flex cDNA Kit (Quanta Biosciences). Random primers or Gene-specific primers were used in the RT reaction. To determine whether lncRNA candidates are polyadenylated, cDNA with oligo dT primer was also prepared.

Total RNA for microarray was lysed by using a protocol combining TRIzol Reagent and RNeasy Kit (Qiagen). Next, total RNA was resuspended in RNase-free water. The quality of the RNA was analyzed by Bioanalyzer (Agilent Technologies, Santa Clara, CA, USA). Only RNAs with RNA Integrity Number (RIN) >9.5 were used for subsequent experiments.

### 2.7. Microarrays

Purified total RNAs after miR-122 with or without HCV treatment were amplified and Cy3-labeled using the LIRAK kit (Agilent Technologies) following the kit instructions. Per reaction, 200 ng of total RNA was used. The Cy3-labeled RNA was hybridized overnight to 8 × 60K 60mer oligonucleotide spotted microarray slides (Agilent Technologies, design ID 072363). Hybridization and subsequent washing and drying of the slides were performed following the Agilent hybridization protocol. The dried slides were scanned at 2 µm/pixel resolution using the InnoScan 900 (Innopsys, Carbonne, France). Image analysis was performed with Mapix 6.5.0 software, and calculated values for all spots were saved as GenePix results files. Stored data were evaluated using the R software [31] and the limma package [32] from BioConductor [33]. Mean spot signals were background corrected with an offset of 1 using the NormExp procedure on the negative control spots. The logarithms of the background-corrected values were quantile-normalized [32,34]. The normalized values were then averaged for replicate spots per array. From different probes addressing the same NCBI gene ID, the probe showing the maximum average signal intensity over the samples was used in subsequent analyses. Genes were ranked for differential expression using a moderated *t*-statistic [32]. Pathway analyses were done using gene set tests on the ranks of the *t*-values [32]. Z value was calculated according to formula: Z = (E − $\bar{E}$)/SD, where E is the quantile-normalized log2 signal intensity, $\bar{E}$ is the mean value of E, SD indicates the standard deviation across the samples.

### 2.8. Quantitative Real Time-PCR (qRT-PCR)

qRT-PCR was performed with the PerfeCTa SYBR Green FastMix (Quanta Biosciences) according to the manufacturer’s instructions in the StepOnePlus™ Real-Time PCR System (Applied Biosystems) with the following temperature setting: initial denaturation for 20 s at 95 °C; 40 cycles of subsequent denaturation (3 s at 95 °C) and elongation (30 s at 60 °C); melting curve for 20 min. The secondary products and primer-dimers were excluded via melting curve and agarose gel electrophoresis. The specificity of amplification was verified by the presence of a single peak in the melting curve and also by sequencing (Microsynth SeqLab, Germany). Glyceraldehyde 3-phosphate dehydrogenase (*GAPDH*) mRNA levels were evaluated in all cases as a reference, and other expression results were normalized to *GAPDH*. Amplification efficiencies (E) of each primer pair were calculated using the following formula: E = 10(-1/slope). The E of primers used in this study was within the range of 1.8-2.2 [35]. To calculate the relative RNA levels in cytoplasmic/nuclear fractions, 2^−∆Ct^ was used, where Ct is the threshold cycle number, ∆Ct = Ct of the gene in nucleus-Ct in cytoplasm. The expression fold change compared to control group was obtained using calculation: Fold change = (E_target_)^∆Ct_target_^(control-sample)/^(E_ref_)^∆Ct_ref_^(control-sample)^, where E_target_ and E_ref_ are the respective amplification efficiencies of target genes and reference gene *GAPDH*; ∆Ct = Ct of the control sample - Ct of the treatment sample. The relative expression level of lncRNAs after GmR knockdown was presented as 1000*2^−∆Ct^, ∆Ct = Ct of the target gene - Ct of the reference gene *GAPDH*. The results of all biological replicates (minimum of three) and technical replicates (minimum of two) were used to derive the final data with standard error of the mean (SEM) graphed as error bars. 

### 2.9. Immunofluorescence

One day before transfection, coverslips were heated in pure Ethanol and covered for 30 min with 0.1 mg/mL Poly-L-Lysin (30000-70000). Two days after transfection of HCV full-length Jc1 genomes, cells were washed with PBS and fixed with 4% paraformaldehyde for 10 min. Cells were washed again 3 times (×) with ice cold PBS, permeabilized with cold acetone for 10 min at −20 °C and again washed. Then, cells were incubated with 1% BSA, 22.5 mg/mL glycine in PBST (1 × PBS, 0.5% Tween 20) (Glycin-PBST) for 10 min. For staining, cells were incubated with a 1:500 dilution of Anti-HCV NS3 antibody (8 G-2, Abcam) in 1% BSA for 1 h at room temperature. Cells were washed 3 × with Glycin-PBST and then incubated with a 1:200 dilution of the secondary antibody (goat anti-mouse IgG1, Alexa Fluor® 488 conjugate) for 1 h at 37 °C in the dark. Cells were again washed 3 × with Glycin-PBST, incubated with Fluoroshield Mounting Medium With DAPI(Abcam)for 5 min. Fluorescent images were obtained with a fluorescent microscope (Olympus).

### 2.10. Western Blot

Cell pellets for western blots were lysed in 200 µL buffer (25 mM Tris-HCl (pH 7.5), 150 mM KCl, 2 mM EDTA (pH 7.5), 0.5 mM DTT, 0.5% NP-40). Following the pelleting of cell debris, 10 µL protein extracts were mixed with SDS loading buffer, denatured at 95 °C for 10 min, and subjected to 12% SDS-polyacrylamide gel electrophoresis. Next, proteins were transferred onto a PVDF membrane (Immobilon). Membranes were blocked with 7.5% milk in TBST for 1 h and incubated with monoclonal antibodies against *GAPDH* diluted 1:15000 (clone GAPDH-71.1, Sigma-Aldrich), Anti-HCV NS3 antibody 8G-2 (Abcam) diluted 1:500. After washing, membranes were incubated for another 1 h with a secondary goat-anti-mouse IgG HOR antibody conjugated with peroxidase diluted 1:40000 (Sigma-Aldrich). Western blots were developed with SuperSignal West Femto Chemiluminescent substrate (Pierce). The quantification of protein bands from western blotting films was performed by using Image J (NIH) (https://imagej.nih.gov/ij/index.html). The expression level was presented as IntDen ratio of each NS3 band relative to each *GAPDH* band.

### 2.11. Protein-Coding Potential

The features of lncRNA candidates, including the reference sequence, the length, Gene symbol and located chromosome of these lncRNAs were collected from NCBI (https://www.ncbi.nlm.nih.gov/) and are listed in Table 3. The names used in this study were based on LNCipedia gene ID or HGNC Gene Symbol. 

Coding potential of lncRNA candidates was evaluated by Open reading frame Finder (https://www.ncbi.nlm.nih.gov/orffinder/), and by searching the LNCipedia 5.2 (http://www.lncipedia.org) for the presence of our candidates in the Pride proteomics database and the Lee lists of novel coding RNAs or Bazzini lists of lncRNAs containing small open reading frames (smORFS) obtained in ribosome profiling experiments. The evaluation of our candidates by Phylogenetic Codon Substitution Frequencies (PhyloCSF) and the coding potential assessment tool (CPAT) were also included [29,36]. Results from LNCipedia are listed in Table 4. 

### 2.12. Statistical Analysis

The graphs showed mean and standard error of mean (Mean ± SEM) of at least three independent experiments. SEM is represented by error bar. Comparisons between groups were performed using two-tailed Student’s *t*-test by GraphPad. *p*-values lower than 0.05 were considered with statistical significance. * denotes *p* ≤ 0.05, ** *p* ≤ 0.01, *** *p* ≤ 0.001, and **** *p* ≤ 0.0001. 

## 3. Results

### 3.1. Identification of lncRNAs Deregulated by HCV Replication

**Figure 1:**
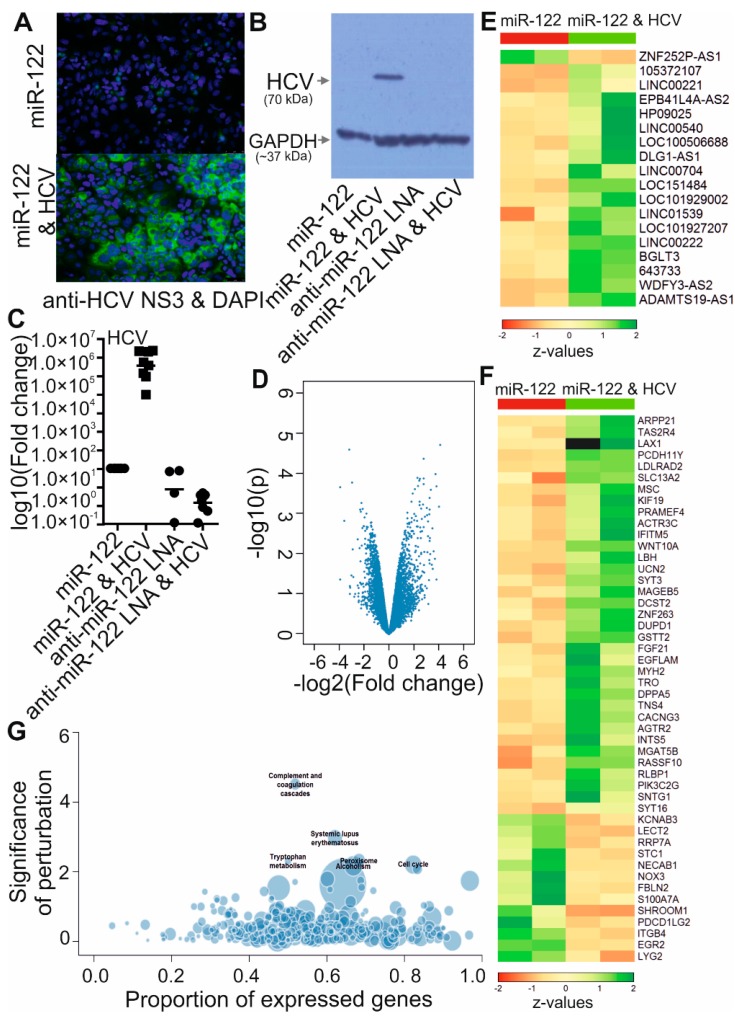
Transcriptome analysis of HCV-deregulated genes in Huh-7.5 cells. HCV NS3 protein level was detected by immunofluorescence (**A**) and western blotting (**B**) 6 days after transfection of HCV full length RNA and treatment with miR-122 duplex or miR-122 as described (Section 2.5). Treatment with anti-miR-122 LNA mixmer alone or with HCV was also performed. qRT-PCR was performed to check the HCV RNA targeting the NS3 coding region (**C**). *GAPDH* was used as a reference gene for normalization. * *p* < 0.05. Total RNA was isolated from Huh-7.5 cells with above treatments in two independent experiments. These RNAs were used for microarray experiments. Comparison of expression levels of sequences from HCV infected cells to uninfected cells was carried out. The volcano plot shows the results for all genes (**D**). Deregulated lncRNAs (**E**) and protein-coding genes (**F**) with fold change > 4 and *p* ˂ 0.01 (log fold change > 2, log_10_P value > 2) are shown in the heatmap. Z value was calculated. The color scale is shown at the bottom. Information about lncRNAs is listed in Table 3. The bubble plot shows enriched KEGG pathway annotation of differentially expressed genes (**G**).

To identify deregulated lncRNAs induced by HCV replication, we carried out a gene expression microarray assay. Huh-7.5 cells were electroporated either with miR-122 only or with miR-122 plus HCV full-length genomic RNA, and then left for 6 days. Since Huh-7.5 cells contains somewhat lower levels of miR-122 than primary hepatocytes, miR-122 transfection was performed one day before and three days after the electroporation of HCV RNA to mimic a high level of miR-122, which is essential for HCV replication [37,38,39,40]. The 6-day duration of HCV replication was chosen to analyze the changes in expression levels of low abundance lncRNAs under conditions similar to long-term infection. A large fraction of the cells contained replicating virus at the harvest day, as evaluated by immunofluorescence and western blotting against HCV protein (Figure 1A,B) and by qRT-PCR targeting HCV RNA in the NS3 coding region (Figure 1C). 

RNA samples from two independent biological replicates were used to hybridize an array in the Human G3 v3 Microarray Kit. Analysis of the expression changes in transcripts showed 68 deregulated genes with fold changes > 4 and *p* ˂ 0.01 (log fold change > 2, log_10_P value > 2) in HCV treated cells compared to control cells (Figure 1D). They were involved in different cellular process including immune response, amino acid metabolism, cell cycle, lipid homeostasis and alcoholism according to the KEGG analysis (Figure 1G). Eighteen putative lncRNAs and 48 protein coding mRNAs showed a significant change of expression level in Huh-7.5 cells upon HCV replication (Figure 1E,F; fold change > 4, *p* ˂ 0.01). 

### 3.2. HCV Replication Increases the Expression of Four lncRNAs

**Figure 2:**
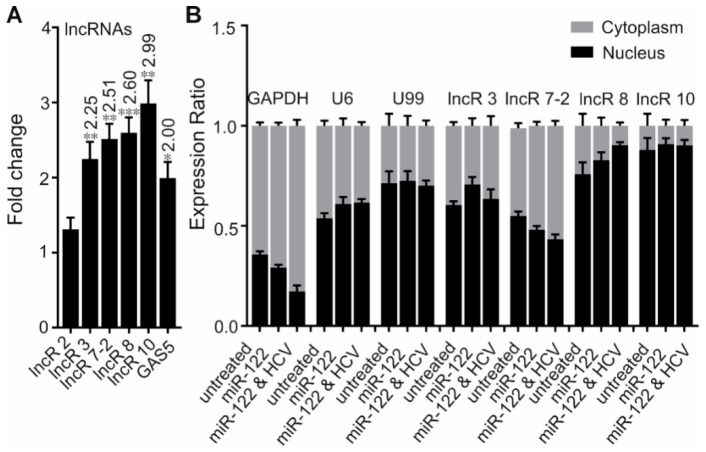
Four selected lncRNA candidates are upregulated by HCV. (**A**) The differential expression of five selected lncRNAs was confirmed by qRT-PCR 6 days after HCV transfection. GAS5 was used as a positive control. Data were normalized to *GAPDH*. Fold changes of lncRNA expression comparing HCV-treated cells to control cells are indicated at the top of each bar when statistically significant. Experiments were repeated a minimum of three times, with at least two replicates each time, and are represented as mean ± SEM. * *p* ≤ 0.05, ** *p* ≤ 0.01, and *** *p* ≤ 0.001. (**B**) The subcellular localization of lncR 3, lncR 7-2, lncR 8, and lncR 10 was measured by qRT–PCR after cell fractionation. RNA was collected from untreated cells, miR-122 treated, and miR-122 plus HCV RNA treated Huh-7.5 cells. *GAPDH* was used as cytoplasmic control. *U6* and *U99* were used as nuclear controls. Percentage of nuclear and cytoplasmic RNA levels were calculated depending on 2^−∆Ct^, where ∆Ct = Ct of the gene in nucleus - Ct in cytoplasm. The graph shows the average of at least three independent experiments, and represents data as mean ± SEM.

In total, 11 lncRNAs (here renamed to lncR 1-10, nine upregulated and one downregulated in response to HCV replication; whereby lncR 7 has two variants, labeled as lncR 7-1 & 2) were selected for further investigation. To our knowledge, none of them had been functionally studied to date. Changes of the transcript levels observed in HCV replicating samples versus control were verified by qRT-PCR. *GAS5* [22] was used as a positive control. Two variants of lncR 7 have 70 bp difference in sequence, they were amplified separately by variant specific primers (see Table 1). Six candidates were discarded due to failed (lncR 5, 6) or poor amplification (lncR 1, 4, 7-1, 9), which is mainly caused by their very low expression levels. A consistent result between the data of the qRT-PCR and microarray analysis was observed for lncR 3, 7-2, and 8 (Figure 2A). Samples after HCV or mock treatment without adding ectopic miR-122 were also prepared. Similar upregulation of lncR 8 was also observed in samples added ectopic miR-122 but only containing endogenous miR-122 (Figure S2B). LncR 2 expression was not altered by HCV replication, while lncR 10 was upregulated (Figure 2A), showing a result opposite to the microarrays (Figure 2E). In this context, it is interesting to note that lncR 10 was reported to be upregulated in hepatocellular carcinoma (HCC) tissues compared to adjacent non-tumor tissues in another study [41]. Concerning this possible link between HCV infection and HCC, we therefore also proceeded with lncR 10. 

### 3.3. Low Protein Coding Potential and Subcellular Localization of lncRNAs 

ORF Finder (NCBI) was used to determine all possible ORFs in four candidate lncRNAs. Putative ORFs longer than 100 amino acids (aa), which was set as a noncoding threshold, were screened for the presence of Kozak sequences (A/GCCACC or A/GCC) at the initiation codon. No results indicating coding capacity for these four lncRNA candidates were obtained (Data not shown). LncR 8/*Lnc-ITM2C-1* was predicted as a coding gene according to CPAT (69.31%) but not interpreted as coding RNA according to PhyloCSF (-112.1426), and it was also not present in the PRIDE archive, and not in the Lee and the Bazzini coding RNA lists (Table 4). LncR 3, 7-2, and 10 were all described as non-coding RNA in LNCipedia, indicating a very low probability for coding (Table 4).

The preference of nuclear or cytoplasmic location can give clues for the function of a lncRNA [14,41,42]. To gain insight into the potential roles of the lncRNAs, we evaluated the subcellular localization of lncRs 3, 7-2, 8, and 10 in untreated Huh-7.5 cells and Huh-7.5 cells treated with miR-122 alone or miR-122 plus HCV. As expected, *GAPDH* reference transcripts accumulate preferentially in the cytoplasm in treated or untreated Huh-7.5 cells (Figure 2B). In contrast, more *U6* RNA was found to be in the nucleus compared to cytoplasm. The relatively high ratio of *U6* reference transcripts in the cytoplasm may be due to a leakage during nucleus/cytoplasm fractionation, which was also found in a previous study [43]. Therefore, we used *U99* RNA as an additional control; *U99* RNA was more localized in the nucleus. Importantly, lncR 8 and 10 were dominantly accumulated in the nucleus, while LncR 3 and 7-2 were found in both fractions. The nuclear enrichment of lncR 8 and 10 further confirmed their noncoding nature. No obvious difference in subcellular translocation due to the treatment with miR-122 or with HCV was observed. Thus, the different subcellular locations of our lncRNA candidates indicate different function and regulation mechanisms. In particular, lncR 8 (which is further analyzed below) is localized in the nucleus.

### 3.4. LncR 8/Lnc-ITM2C-1 Favors HCV Viral Replication

**Figure 3:**
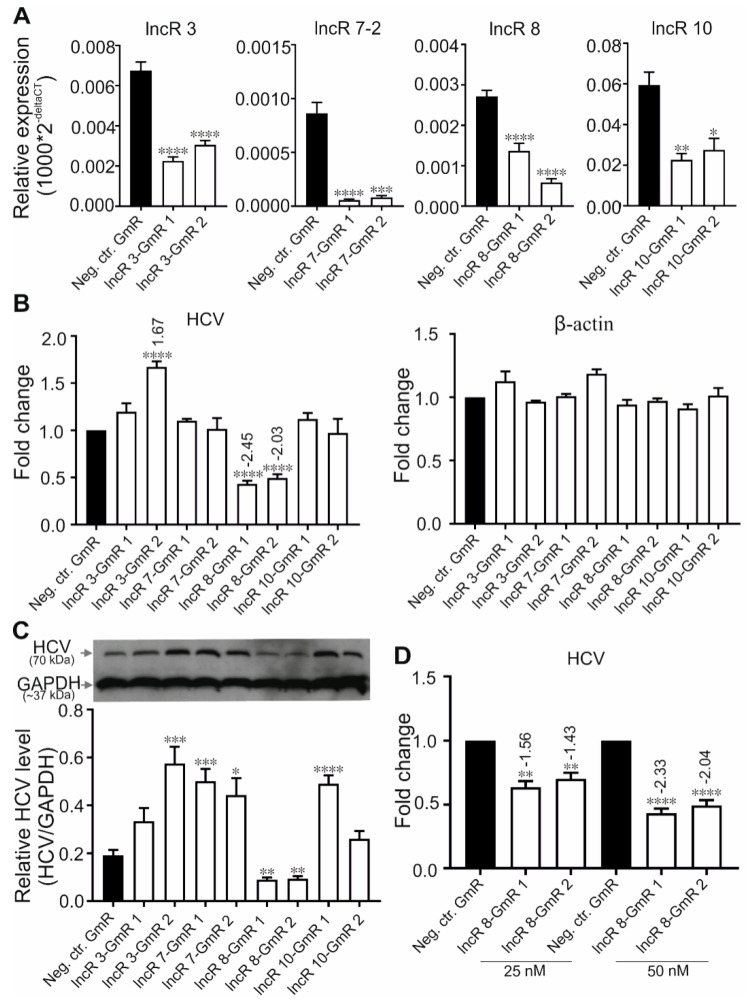
Suppression of lncR 8 inhibits HCV replication. (**A**)The efficiency of GmRs suppressing lncRs 3, 7-2, 8, and 10 was determined by qRT-PCR in Huh-7.5 cells. One day prior to HCV treatment, GmRs targeting lncRNA candidates and Neg. ctr.GmR were transfected in Huh-7.5 cells. Cells were collected at 48 h post HCV transfection. qRT-PCR data of targeted genes was normalized to *GAPDH*. The data are shown as the mean ± SEM of at least three independent experiments. * *p*≤ 0.05, ** *p* ≤ 0.01, *** *p* ≤ 0.001, and **** *p* ≤ 0.0001.HCV RNA and protein level after GmR treatment were detected by qRT-PCR (**B**) and western blotting (**C**). HCV RNA level after different concentration of lncR 8-GmR treatment was detected by qRT-PCR (**D**). qRT-PCR data of HCV RNA in (**B**) was normalized to *GAPDH*. Another housekeeping gene *β-actin* was used as a negative control. Altered HCV level with significance are marked with numbers at the top of the bar. The numbers indicate fold changes of HCV RNA expression after GmRs treatment, where positive numbers mean upregulation, negative numbers mean downregulation. The upper panel in (**C**) is representative western blot of HCV NS3 protein. The lower panel is the quantification of protein bands from western blot was performed by using Image J (NIH). IntDen ratio of each NS3 band relative to each GAPDH band was presented. The data are shown as the mean ± SEM of at least three independent experiments.

To evaluate the role of the lncRNAs in viral replication, we depleted lncRNAs from cells with GapmeRs (GmRs) independently targeting two different sites in the respective lncRNA to minimize off-target effects. LNA™ longRNA GapmeRs are single-stranded antisense oligonucleotides that contain a central block of deoxynucleotide monomers, flanked by locked nucleic acid (LNA) stretches for strong target binding and nuclease resistance. The central DNA block induces RNase H mediated degradation of the target RNA and can be used for knockdown of lncRNA and mRNA in cell cultures and even in animal models [44,45]. GmRs are effective at degrading both nuclear and cytoplasmic lncRNAs [44]. Cells were transfected with the specific GmRs or with negative control GmR (Neg. ctr. GmR, which contains a randomized targeting sequence) one day prior to transfection with HCV RNA and collected at indicated time points. The Neg. ctr. GmR transfection serves to level out unspecific effects that may be caused by the transfected GmRs in general. To display GmR binding specificity in the genome, CLUSTAL and NBLAST analyses were done. The results show that the Neg. ctr. GmR does not bind specifically to any target in the human transcriptome, and all GapmeRs specific for lncRNAs used in this study are very specific for their genuine targets, except that lncR 10-GmR1 has a single off-target with only 1 nt difference (Figure S1).The suppression of targeting lncRNAs 48 h after HCV transfection was examined by qRT-PCR (Figure 3A). Reference gene *GAPDH* was used for normalization. Since qRT-PCR detection of expression levels may significantly differ when targeting the 5′ or the 3′ side of the GmR target sequences [46], two different sets of primers targeting both sides of lncRNA sequence were checked and compared (Data not shown). Primers amplifying 5′ side sequence of lncRs 3 and 7-2, and 3′ side primers for lncRs 8 and 10 were used to determine GmR effects in this study. Both GmRs against lncRNAs functioned efficiently (Figure 3A). 

HCV RNA level after lncRNA knockdown was examined by qRT-PCR. Since *GAPDH* was used for ‘well-to-well’ normalization within each experiment to correct for slight variations in samples, another house-keeping gene *β-actin* was used as a negative control gene, which is not supposed to be affected by the treatment (Figure 3B). *β-actin* did not show changes due to treatment. Two GmRs targeting lncR 3 stimulated HCV RNA expression, but the stimulation by lncR3-GmR 1 was not statistically significant, whereas lncR3-GmR 2 significantly induced upregulation of HCV protein level (Figure 3C). No change of HCV RNA levels was observed after the silencing of lncRs 7-2 and 10 (Figure 3B). However, HCV protein level was upregulated in lncR 7 knockdown samples and in lncR 10-GmR 1 treated samples (Figure 3C). Considering the extremely low level of lncR 7-1 in Huh-7.5 cells that was not detected by qRT-PCR in our study, the upregulated HCV protein after lncR 7 knockdown was believed to be mainly the effect of suppression of variant 2 by GmRs that target both variants. These results pointed out a negative regulation of HCV translation, but not replication, by lncR 7-2. Given the inconsistent effects caused by two GmRs targeting lncRs 3 and 10, we cannot exclude that the changes of HCV expression is caused by off-target effect of lncR3-GmR 2 and lncR 10-GmR 1. In contrast, viral RNA and viral protein were both decreased after lncR 8 suppression (Figure 3B,C). Based on these results, we learned that lncR 7-2 is a negative regulator of HCV, while lncR 8 supports HCV replication. Therefore, we focused on lncR 8 in the following. When higher concentration of lncR 8-GmR was added in cells, HCV RNA expression showed a stronger decrease, further confirming a correlation between lncR 8 level and HCV replication level (Figure 3D).

### 3.5. LncR 8/Lnc-ITM2C-1 Is a Short-Term Cis-Acting Regulator of Its Neighbor GPR55

**Figure 4:**
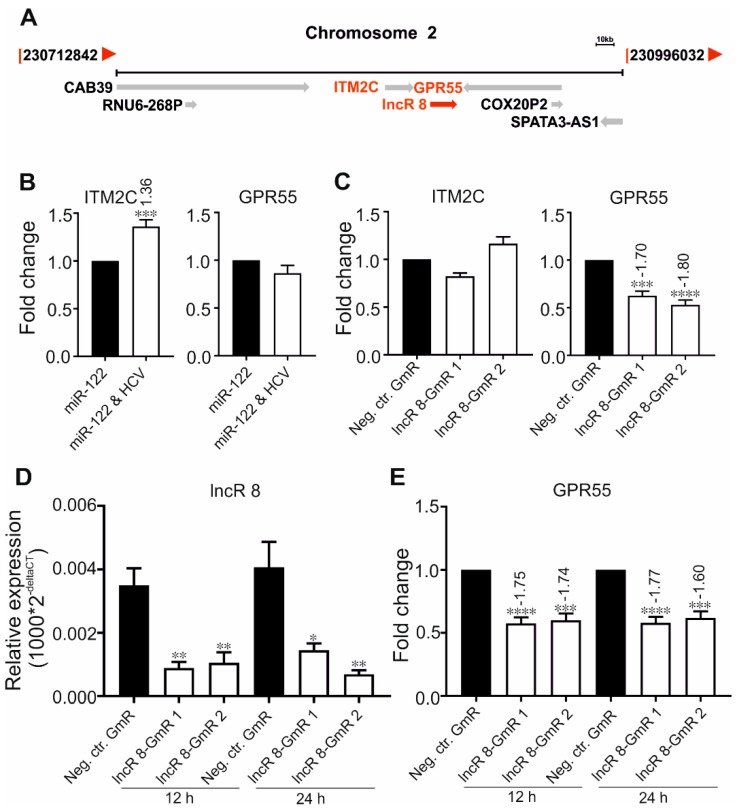
LncR 8 positively regulates neighboring gene *GPR55*. (**A**) Genomic location of lncR 8 and the relationship with the genes encoding *ITM2C* (Integral Membrane Protein 2C) and *GPR55* (G protein-coupled receptor 55). (**B**) *ITM2C* and *GPR55* expression in HCV-transfected samples and controls was measured 6 days after transfection and compared. Cells were treated as described in Figure 1. qRT-PCR data was normalized to *GAPDH*. The data are shown as the mean ± SEM of at least three independent experiments. *** *p* ≤ 0.001, and **** *p* ≤ 0.0001. Huh-7.5 cells were treated with Neg. ctr. GmR and lncR8-GmRs one day prior to HCV transfection. Cells were collected at indicated time points post HCV transfection. (**C**) *ITM2C* and *GPR55* expression level at 2 days after lncR 8-GmRs and HCV transfection. qRT-PCR data was normalized to *GAPDH*. The knockdown of lncR 8 (**D**) and the effect on neighboring gene *GPR55* (**E**) was examined at these early time points by qRT-PCR. To illustrate the differences in basal expression levels, the values are shown relative to *GAPDH*, expressed as 2^−ΔCt^. The data are shown as the mean ± SEM of at least three independent experiments. * *p* ≤ 0.05, ** *p* ≤ 0.01, *** *p* ≤ 0.001, and **** *p* ≤ 0.0001.

Previous studies showed that lncRNAs can regulate neighboring genes [47]. The genes for Integral Membrane Protein 2C (*ITM2C*) and G protein-coupled receptor 55 (*GPR55*) are within 10 kb distance of lncR 8 in the genome (Figure 4A). To gain further insight into the regulatory mechanism of lncR 8, we evaluated the expression of neighboring genes at 6 days after HCV RNA transfection as well as 2 days after lncR 8 suppression in Huh-7.5 cells. Though *ITM2C* was identified with high expression level in HCV-induced HCC tissues compared to HCV-induced HCC non-tumor liver tissues [48], we found only a very mild increase of *ITM2C* induced by HCV replication in Huh-7.5 cells (Figure 4B). Furthermore, no change of *ITM2C* mRNA levels was observed after lncR 8 knockdown (Figure 4C). This rules out a *cis*-regulatory activity of lncR 8 on *ITM2C* during HCV replication. 

The mRNA expression of the other neighboring gene *GPR55* was not significantly altered after 6 days of HCV replication (Figure 4B). HCV triggered lncR 8 expression but did not change *GPR55* expression after 6 days, on first glance arguing against a correlation between lncR 8 and *GPR55*. However, lncR 8 knockdown suppressed *GPR55* expression at 48 h post GmR and HCV treatment in Huh-7.5 cells (Figure 4C). In addition, samples after lncR 8-GmRs treatment and further HCV transfection for 12 h and 24 h were examined. Downregulation of *GPR55* mRNA levels was also observed at these early time points when lncR 8 was suppressed efficiently (Figure 4D,E). These data strongly indicate a positive regulation of *GPR55* by lncR 8 in HCV transfected cells. Considering the different time length in the experimental settings, we hypothesize that lncR 8 controls *GPR55* at early times (12, 24, 48 h), while at late times (6 d) the effect of lncR 8 on *GPR55* expression may be counteracted by other mechanisms. Taken together, the positive effect of lncR 8 on *GPR55* by *cis*-regulation may act only within a short time period after HCV replication. 

### 3.6. LncR 8/Lnc-ITM2C-1 Is a Negative regulator of the Antiviral Response

**Figure 5:**
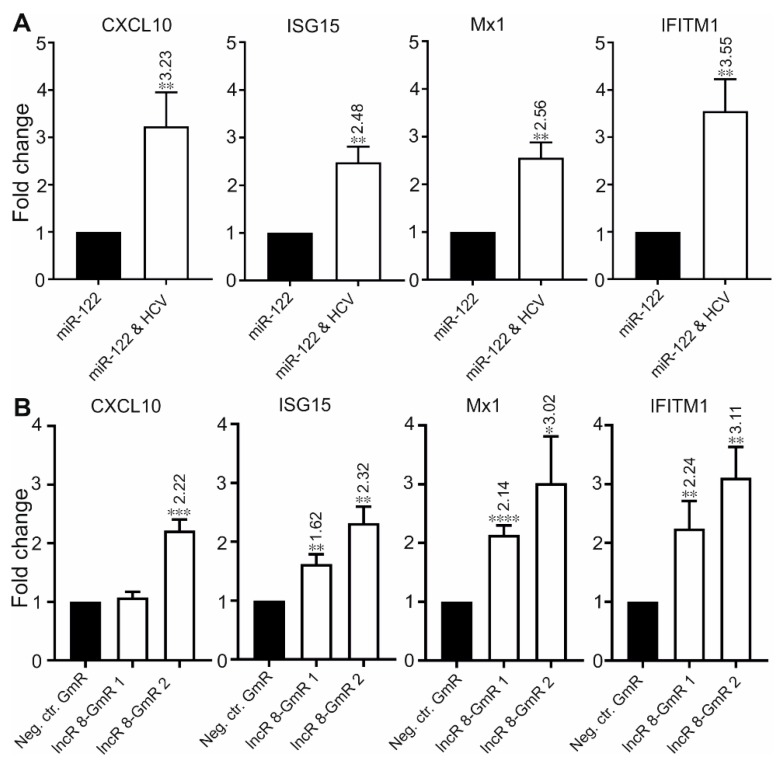
LncR 8 negatively regulates ISGs expression. (**A**) Indicated ISGs expression in samples treated as described in Figure 1 were measured 6 days after HCV transfection. qRT-PCR data was normalized to *GAPDH*. The data are shown as the mean ± SEM of at least three independent experiments. * *p* ≤ 0.05, ** *p* ≤ 0.01, *** *p* ≤ 0.001, and **** *p* ≤ 0.0001. (**B**) ISGs expression level at 48 h after GmRs and HCV transfection were measured. Cells were treated in the same condition as described in Figure 3A. qRT-PCR data was normalized to *GAPDH*. The data are shown as the mean ± SEM of at least three independent experiments.

**Figure 6:**
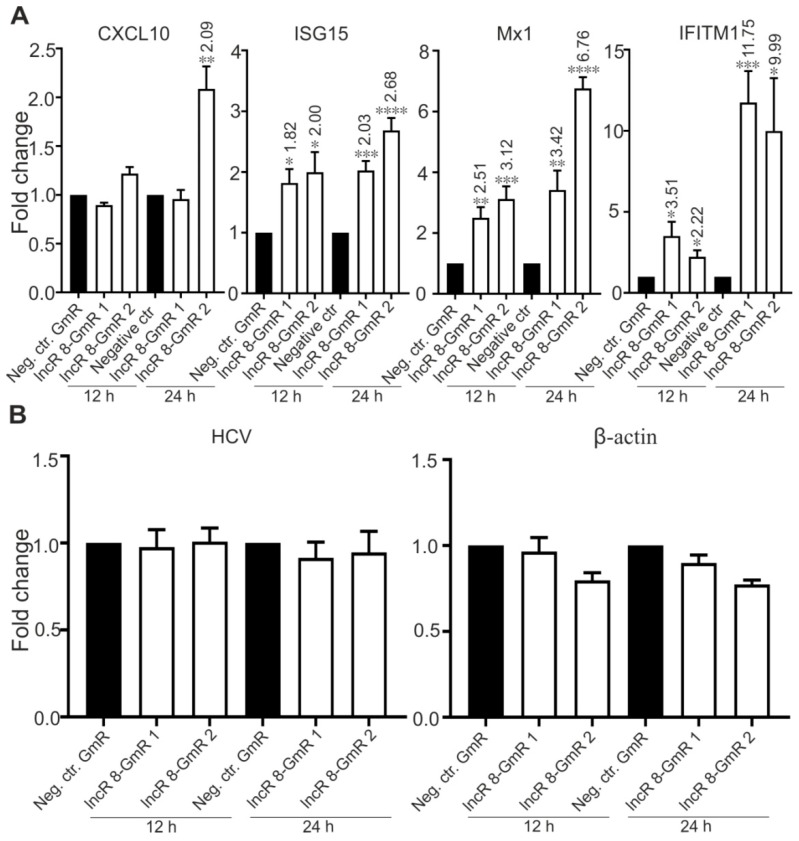
Regulation of ISGs by lncR 8 is earlier than regulation of HCV.ISGs expression level (**A**) and HCV level (**B**) at early time points (12 h and 24 h) were examined by qRT-PCR in the samples described in Figure 4. *GAPDH* was used to normalize. Negative control gene *β-actin* was not altered by lncR 8 knockdown (**B**). The data are shown as the mean ± SEM of at least three independent experiments. * *p*≤ 0.05, ** *p* ≤ 0.01, *** *p* ≤ 0.001, and **** *p* ≤ 0.0001.

Several lncRNAs were proven to affect HCV replication by regulating the interferon response [13,15,25,26,29,49]. To investigate this possibility for lncR 8, we examined the expression levels of four ISGs, *CXCL10*, *ISG15*, *Mx1*, and *IFITM1*, which are involved in immune responses against HCV [14]. In accordance with previous studies, these four ISGs showed increased expression levels after 6 days of HCV replication in the presence of endogenous plus ectopically added miR-122 (Figure 5A) or without ectopically added but only with endogenous miR-122 in the Huh-7.5 cells (Figure S2D), showing a successfully induced immune response after HCV replication in Huh-7.5 cells. Furthermore, 48 h post HCV transfection followed by lncR 8 silencing for 24 h in Huh-7.5 cells, significant increases of ISG levels compared to control cells were also observed (Figure 5B), except for *CXCL10* when lncR 8-GmR 1 was used. This indicates that lncR 8 is a negative regulator of ISGs. In addition, following 24 h of lncR 8-GmR incubation, samples were obtained at 12 and 24 h post HCV transfection. Under these conditions, ISGs, except for *CXCL10*, were upregulated by lncR 8 silencing (Figure 6A), while HCV RNA level was not altered (Figure 6B). Since *GAPDH* was used for data normalization, an additional house-keeping gene (*β-actin*) was used as a negative control target, which was not changed due to lncR 8-GmR treatment compared to negative control cells (Figure 6B). These results show that the upregulation of ISGs expression by lncR 8 suppression occurs earlier than the decrease of HCV RNA levels. Taken together, our data indicate that the suppression of HCV may be the result of ISGs′ increase induced by lncR 8 knockdown.

### 3.7. LncR 8/Lnc-ITM2C-1 Is Upregulated by HCVcc Infection and Facilitates HCV Infection

**Figure 7:**
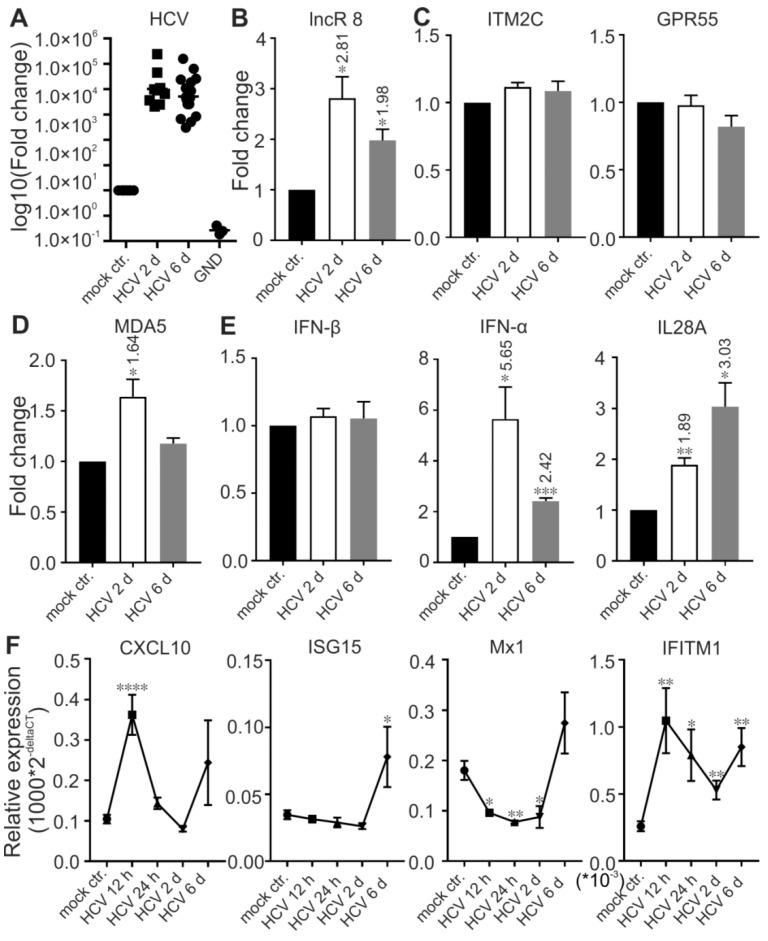
lncR 8 and IFITM1 are upregulated by HCV infection. The expression of HCV(**A**), lncR 8 (**B**), neighboring genes (**C**), *MDA5* (**D**), and indicated IFNs (**E**) were confirmed by qRT-PCR at 2- or 6-days post HCV infection. NS3 level was also tested in cells infected with a replication defective mutant version of the HCV genome (NS5B replicase inactivating "GND" mutation). Expression of ISGs (**F**) was also detected at earlier time points post HCV infection. Data were normalized to *GAPDH*. Fold changes of mRNA expression comparing HCV infected cells to control cells are indicated at the top of each bar when statistically significant. Experiments were repeated a minimum of three times, with at least two replicates each time, and are represented as mean ± SEM. * *p* ≤ 0.05, ** *p* ≤ 0.01, *** *p* ≤ 0.001, and **** *p* ≤ 0.0001.

To understand the function of lncR 8 in real HCV infection, HCV infectious particles were prepared and used to infect Huh-7.5 cells at MOI of 0.3 for 12 h, 24 h,2 d, and 6 d. lncR 8 and *MDA5* were upregulated by HCV infection for 2 d. However, this effect disappears at 6 d post infection (Figure 7B,D). *IFN-β* was not changed due to HCV infection, while another type I IFN, *IFN-α*, and type III IFN, *IL28A*, were upregulated at 2 d and 6 d post infection (Figure 7E). *ISG15* and *IFITM1* were upregulated at 6 d post HCV infection (Figure 7F), similar to that we observed after HCV RNA transfection (Figure 5A). At earlier time, *IFITM1* expression level was also increased due to HCV infection, while *CXCL10* was only induced at 12 h post HCV infection, and *Mx1* was downregulated after HCV infection for 12 h, 24 h, and 2 d but increased only after 6 d.

**Figure 8:**
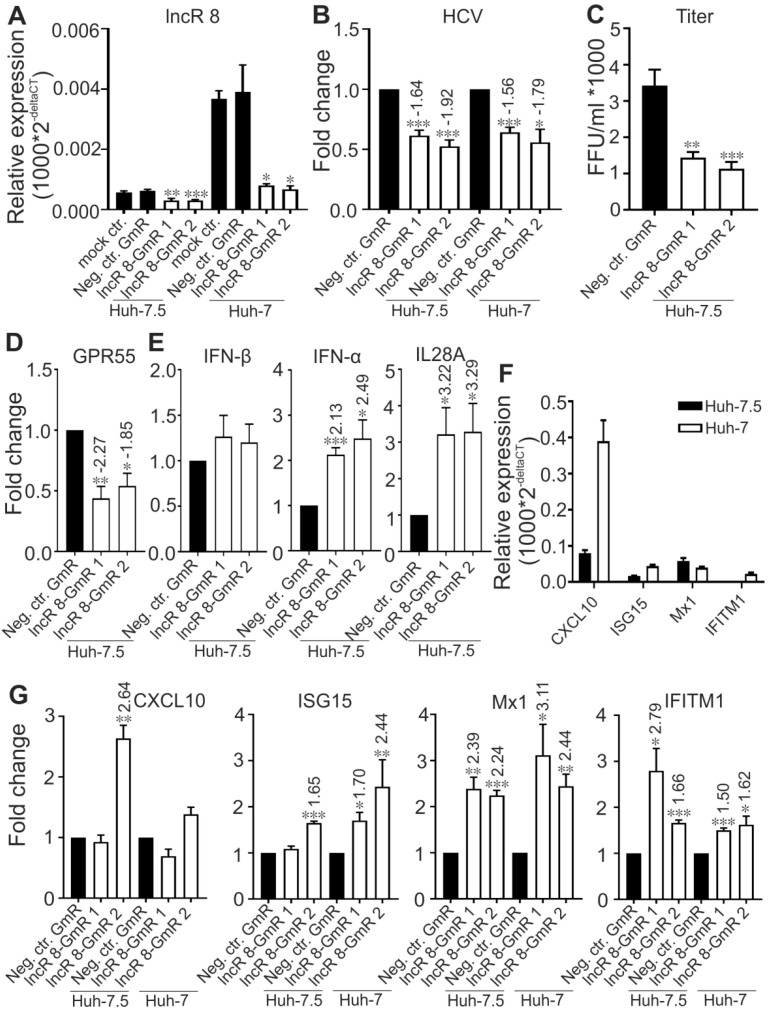
Suppression of lncR 8 inhibits HCV infection and promotes ISGs expression in both Huh-7.5 and Huh-7 cells. (**A**)The efficiency of GmRs suppressing lncR 8 was determined by qRT-PCR in Huh-7.5 and Huh-7 cells. One day prior to HCV treatment, GmRs targeting lncRNA candidates and Neg. ctr. GmR were transfected in cells. Cells treated with mock ctr. (without GmRs) were also detected to show that no unspecific influence was induced by Neg. ctr. GmR on lncR 8. Cells were collected at 48 h post HCV infection. qRT-PCR data of targeted genes was normalized to *GAPDH*. The data are shown as the mean ± SEM of at least three independent experiments. * *p* ≤ 0.05, ** *p* ≤ 0.01, and *** *p* ≤ 0.001. HCV RNA and virus titer after GmR treatment were detected by qRT-PCR (**B**) and focus-forming units (FFU) assay (**C**). *GPR55* (**D**), IFNs (**E**), and ISGs (**G**) expression were also detected. Basal relative expression level of ISGs expression in Huh-7.5 and Huh-7 cells was shown in (**F**). Altered mRNA expressions with significance are marked with numbers at the top of the bar. The numbers indicate fold changes of expression after GmRs treatment, where positive numbers mean upregulation, negative numbers mean downregulation.

**Figure 9:**
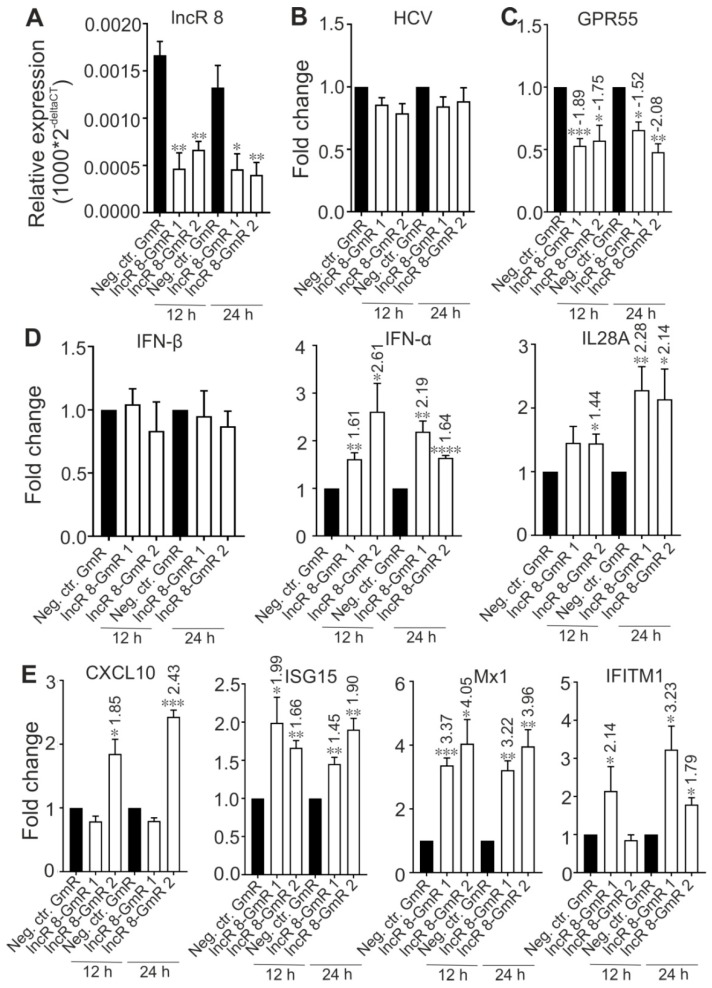
Suppression of lncR 8 promotes ISGs and inhibits *GPR55* expression at early time. (**A**) The efficiency of GmRs suppressing lncR 8 was determined by qRT-PCR in Huh-7.5 cells. HCV RNA (**B**), *GPR55* (**C**), indicated IFNs (**D**), and ISGs (**E**) after GmR treatment were detected by qRT-PCR. One day prior to HCV treatment, GmRs targeting lncRNA candidates and Neg. ctr. GmR were transfected in Huh-7.5 cells. Cells were collected at 12 h and 24 h post HCV infection. qRT-PCR data of targeted genes was normalized to *GAPDH*. The data are shown as the mean ± SEM of at least three independent experiments. * *p* ≤ 0.05, ** *p* ≤ 0.01, *** *p* ≤ 0.001, and **** *p* ≤ 0.0001. Altered mRNA level with significance are marked with numbers at the top of the bar. The numbers indicate fold changes of mRNA expression after GmRs treatment, where positive numbers mean upregulation, negative numbers mean downregulation.

In cells first treated with GmR for 24 h and then infected with HCV for 48 h, HCV viral genome and titer was decreased in samples when lncR 8 was decreased by GmRs (Figure 8A–C). Representative ISGs were upregulated in HCV infected cells with lncR8-GmR treatment compared to negative control (Figure 8G). Similar upregulation of ISGs and downregulation of HCV NS3 expression by lncR 8 knockdown were also observed in Huh-7 cells (Figure 8B,G). Similar to the results observed when treated with HCV in vitro transcribed RNA (Figure 6A), the upregulation of ISGs expression was observed early at 12 h post GmR treatment and HCV infection (Figure 9E), while the downregulation of HCV RNA only occurred at 48 h, indicating that the downregulation on ISGs expression by lncR 8 happened earlier than the downregulation on HCV infection. *GPR55* was downregulated by lncR 8 knockdown at all the time points we tested (Figure 8D and Figure 9C), similar to what we observed when cells were transfected with HCV RNA (Figure 4C,E). Though *IFN-β* was not influenced by lncR 8 change at the time points we tested, both *IFN-α* and *IL28A* were upregulated by lncR 8 inhibition in Huh-7.5 cells (Figure 8E and Figure 9D). Taken together, lncR 8 is positively regulated by HCV and has a role in stimulating HCV replication by suppression of interferon responses. 

### 3.8. GPR55 Negatively Regulates ISGs

**Figure 10:**
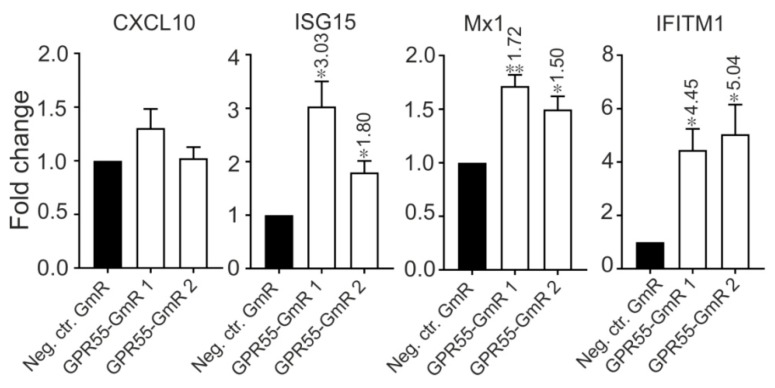
Suppression of *GPR55* promotes several ISGs expression. Two GmRs targeting *GPR55* and Neg. ctr.GmR were transfected in Huh-7.5 cells. Cells were collected at 48 h later and ISGs expression was measured by qRT-PCR. qRT-PCR data was normalized to *GAPDH*. The data are shown as the mean ± SEM of at least three independent experiments. * *p* ≤ 0.05 and ** *p* ≤ 0.01. Altered mRNA level with significance are marked with numbers at the top of the bar. The numbers indicate fold changes of mRNA expression after GmRs treatment.

Since both *GPR55* and ISGs are negatively regulated by lncR 8, it is interesting to know whether there is a correlation between *GPR55* and ISGs. Therefore, *GPR55* expression was inhibited by two different GmRs in Huh-7.5 cells for 48 h. Suppression of *GPR55* promotes the expression of *ISG15*, *Mx1*, and *IFITM1* (Figure 10). This finding provides a possible link between lncR 8 and ISGs expression. 

### 3.9. LncR 8/Lnc-ITM2C-1 Is Induced by polyIC

**Figure 11:**
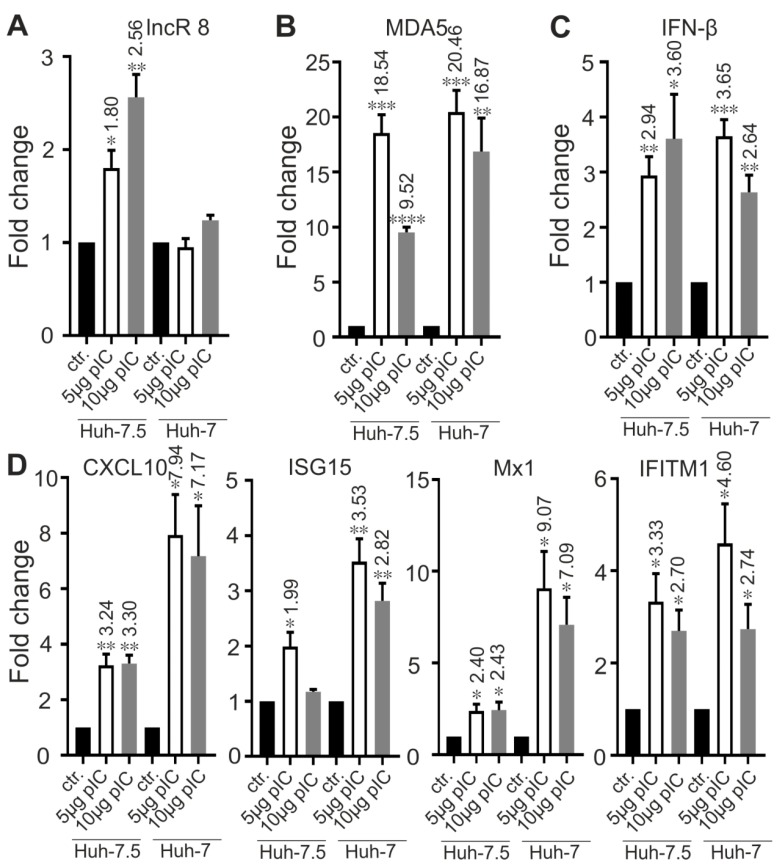
lncR 8 is efficiently induced by poly(I:C) treatment in Huh-7.5 cells but not in Huh-7 cells. Cells were treated with 5ug or 10 ug poly(I:C) (pIC) and collected after 8 h incubation. qRT-PCR data of targeting genes, including lncR 8, *MDA5*, *IFN-β* and ISGs, was normalized to *GAPDH*. The data are shown as the mean ± SEM of at least three independent experiments. * *p* ≤ 0.05, ** *p* ≤ 0.01, *** *p* ≤ 0.001, and **** *p* ≤ 0.0001. Altered mRNA level with significance are marked with numbers at the top of the bar. The numbers indicate fold changes of mRNA expression after treatment, where positive numbers mean upregulation, negative numbers mean downregulation.

Poly(I:C) is a synthetic analog of double-stranded RNA (dsRNA), a molecular pattern associated with viral infection that induces the innate immune response. When Poly(I:C) was used to treat Huh-7.5 and Huh-7 cells, increasing expression level of ISGs were observed in both cells. lncR 8 was upregulated in Huh-7.5 cells (Figure 11A), which suggests that lncR 8 is not induced specifically by HCV. However, lncR 8 was not changed after poly(I:C) treatment in Huh-7 cells (Figure 11A), indicating different responses in these two cells. Poly(I:C) is known to trigger *MDA5*-mediated interferon signaling [50]. *MDA5* and *IFN-β* showed upregulation both in Huh-7.5 and Huh-7 cells treated with poly(I:C) (Figure 11B,C). Relatively lower levels of ISGs were induced by poly(I:C) treatment in Huh-7.5 cells compared to that in Huh-7 cells (Figure 11D), which is consistent with both the general induction of ISGs in the cells and the higher permissiveness of Huh-7.5 cells for HCV replication compared with Huh-7 cells.

**Figure 12:**
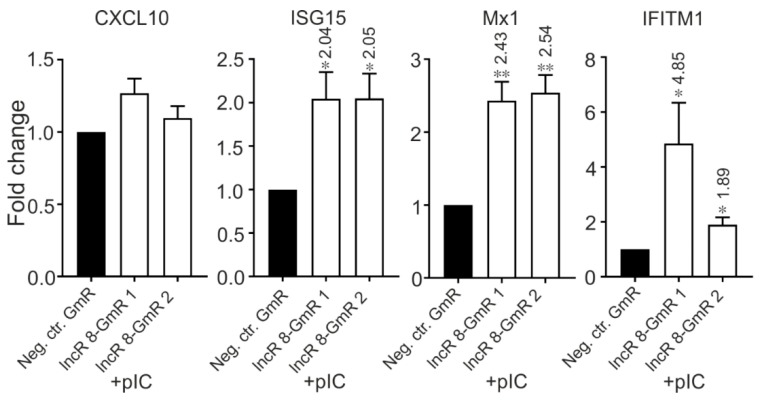
Knockdown of lncR 8 upregulates representative ISGs after poly(I:C) treatment. One day prior to poly(I:C) treatment, GmRs targeting lncR 8 and Neg. ctr. were transfected in Huh-7.5 cells. Cells were treated with 5ug poly(I:C) and collected after 8 h incubation. qRT-PCR data of ISGs was normalized to *GAPDH*. The data are shown as the mean ± SEM of at least three independent experiments. * *p* ≤ 0.05 and **p ≤ 0.01. Altered mRNA level with significance are marked with numbers at the top of the bar. The numbers indicate fold changes of mRNA expression after treatment, where positive numbers mean upregulation, negative numbers mean downregulation.

Importantly, when Huh-7.5 cells were treated with poly(I:C) in combination with lncR 8 GapmeRs for lncR 8 knockdown for 8 h, the ISGs showed increased expression level even compared with control cells with only poly(I:C) treatment (Figure 12). This shows that lncR 8 negatively regulates representative ISGs expression when poly(I:C) was used instead of HCV. 

### 3.10. LncR 8/Lnc-ITM2C-1 Is Downregulated by JAK/STAT Pathway

**Figure 13:**
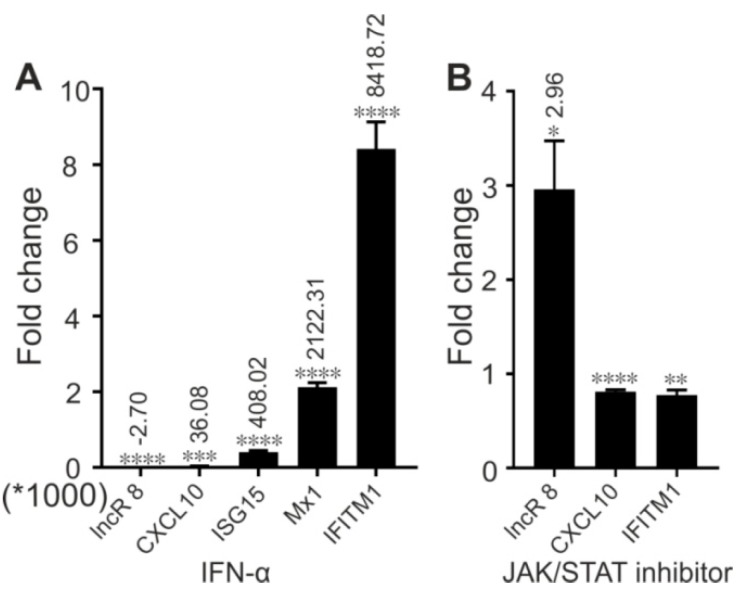
IFN treatment depresses lncR 8 through JAK/STAT pathway. Cells were treated with either mock or IFN-α, and collected after 8 h incubation (**A**). For JAK/STAT inhibition, JAK/STAT inhibitor or mock was added 1 h before IFN treatment (**B**). qRT-PCR data of targeting genes was normalized to *GAPDH*. The data are shown as the mean ± SEM of at least three independent experiments. * *p* ≤ 0.05, ** *p* ≤ 0.01, *** *p* ≤ 0.001, and **** *p* ≤ 0.0001. Altered mRNA level with significance are marked with numbers at the top of the bar. The numbers indicate fold changes of mRNA expression after treatment, where positive numbers mean upregulation, negative numbers mean downregulation.

To learn whether lncR 8 is an ISG, which can be induced by *IFN-α*, like other known lncRNAs, Huh-7.5 cells were treated with *IFN-α* and collected after 8 h. qRT-PCR results show that lncR 8 was negatively regulated by IFN-α (Figure 13A). To determine whether the negative regulation of lncR 8 by *IFN-α* is dependent on JAK-STAT pathway, we treated the Huh-7.5 cells with or without the JAK inhibitor, ruxolitinib, followed by IFN-α treatement. Increased lncR 8 expression was observed when ruxolitinib was added (Figure 13B), indicating that lncR 8 is not an ISG but is negatively regulated by the immune response through the JAK-STAT signaling pathway. 

## 4. Discussion

By using microarray assays, 68 transcripts showed altered expression level upon HCV treatment in Huh-7.5 cells (log fold change > 2, log_10_P value > 2) (Figure 1D). Compared to the lncRNAs identified in the study of Carnero and coworkers [29], the number we obtained is much lower. Surprisingly, among the 68 altered candidates, no overlapping genes were found between these two studies (Carnero et al., 2016) (Figure 1F,G). It is reasonable to believe that the different experimental conditions in these two studies are the major reason for this difference. Consistent with other previous studies, the coding genes we identified as HCV-upregulated (Figure 1G), like wingless-type MMTV integration site family member 10A (*WNT10A*), dual specificity phosphatase and pro isomerase domain containing 1 (*DUPD1*), and fibroblast growth factor 21 (*FGF21*), were previously described to be upregulated by HCV [51,52,53]. Phosphatidylinositol-4-phosphate 3-kinase catalytic subunit type 2 gamma (*PIK3C2G*) is required for HCV replication [54]. Downregulated leukocyte cell-derived chemotaxin 2 (*LECT2*) is a direct target of Wnt/β-catenin signaling in HCC and could be a potential biomarker of HCC in patients [55,56]. Thus, our findings of most coding genes we identified are conformed to previous studies. In contrast, not in line with previous data and also not consistent with our qRT-PCR results [14], ISGs *CXCL10*, *ISG15*, *Mx1*, and *IFITM1* were not found to be deregulated in the microarrays. *MDA5*, which is the main sensor in *RIG-I* defective Huh-7.5 cells [50], was not detected to be differentially regulated in the microarrays (data not shown). Known lncRNAs like GAS5 and EGOT did not show changes due to HCV replication, which is in contrast to previous studies [22,29]. Despite the different experimental settings, the discrepancy between our microarray results with previous studies and our qRT-PCR results can be caused by several other factors. In the first place, low abundancies of lncRNAs could cause high variance of sequencing results [13]. Perhaps even more importantly, low reproducibility of microarray results can occur when experiments are performed by different laboratories, or in the same laboratory but not in a close time period [57]. Adequate number of biological replicates is needed to exclude major sources of variances and exert reliable biological effects [57,58,59]. Except for what mentioned above, different sequencing methods could also lead to largely different results, like the candidates that were obtained by next generation sequencing (NGS) in our recently published paper [60].

Nevertheless, four lncRNA candidates identified by microarray assays, lncRs 3, 7-2, 8, and 10, were verified by qRT-PCR to be HCV-upregulated lncRNAs (Figure 2A). We performed knockdown experiments of lncRNAs to address their effect on HCV replication. Suppressing lncRs 3, 7-2, and 10 did not change the expression of HCV RNA genome, except that lncR 3-GmR 2 induced a moderate increased level of HCV RNA (Figure 3B). Upregulation of HCV protein expression was observed after knockdown of lncR 7 by two GmRs (Figure 3C). These results pointed out an antiviral role of lncR 7-2 by negatively regulating HCV translation, but not replication. In addition, the presence of lncR 7-2 in both nucleus and cytoplasm fractionation (Figure 2B) indicates that lncR 7-2 may regulate mRNA stability or translation, protein transport or post-translational modifications, in addition to regulation of nuclear events [41,42]. Previously, lncR 7-2 was reported to be a direct target of Notch and was positively regulated in T-cell acute lymphoblastic leukemia [61]. Since hepatitis C virus NS3 protein can activate the Notch-signaling pathway [62], upregulated lncR 7-2 may be the result of the activated Notch pathway that was induced by HCV. Further investigation is still needed to decipher the regulation mechanism of lncR 7 by HCV. 

In contrast, lncR 8 suppression with two independent GmRs consistently decreased HCV genomic RNA and protein production (Figure 3B,C), indicating that lncR 8 is required for HCV replication in Huh-7.5 cells. In this study, we further investigated lncR 8. LncRNAs can often regulate their neighboring genes in *cis*, so we examined the expression of nearby genes, *ITM2C* and *GPR55*. *GPR55* was downregulated after lncR 8 suppression at 12, 24, and 48 h in HCV-transfected cells (Figure 4C,E), though this regulation was apparently not maintained at later times since no change of *GPR55* expression was observed when lncR 8 was upregulated 6 days post HCV replication (Figure 2A and Figure 4B). Thus, lncR 8 may regulate *GPR55* by *cis*-regulation only within a short period after HCV replication. Similar results were observed when cells were infected with HCV virus instead of RNA transfection (Figure 7C, Figure 8D and Figure 9C). 

To elucidate the mechanism of proviral activity of lncR 8, ISGs expression were examined after lncR 8 suppression. Surprisingly, two selected ISGs in this study, *Mx1* and *IFITM1*, were upregulated after lncR 8 knockdown in both HCV RNA transfected and virus infected Huh-7.5 cells (Figure 5B and Figure 8G). This suggests that lncR 8 negatively regulates *Mx1* and *IFITM1* during HCV replication and infection. Moreover, the negative effect of lncR 8 on ISGs was also observed at early times when HCV RNA genome abundance was not yet changed (Figure 6A,B and Figure 9E), suggesting that the HCV suppression was probably caused by ISGs increase induced by lncR 8 knockdown. HCV infection of Huh-7.5 cells for 2 days triggered increase of lncR 8 expression compared to uninfected cells, while this regulation was not observed in cells infected with HCV for 6 days. Interestingly, though HCV RNA transfection can induce increased ISGs expression despite of the negative regulation of lncR 8 on ISGs (Figure 5A), HCV infection did not trigger increase of *CXCL10* and *Mx1* in Huh-7.5 cells (Figure 7F). Since transfection sends the HCV RNA directly into the cells, successfully bypassing the membrane recognition and fast immune response induced by membrane receptors, this may lead to longer survival time of HCV replication. Furthermore, it is *RIG-I* but not *MDA5* that recognize in vitro transcribed RNAs in the cytosol [63], while *MDA5* is crucial for interferon production against the infection of picornaviruses [64]. It is worth noting that Huh-7.5 cells have impaired *RIG-I* pathways. Thus, HCV RNA added through transfection failed to be recognized by RIG-I in the Huh-7.5 cells cytosol. Taken together, these findings may explain, on the one hand, the presence of lncR 8 induction in Huh-7.5 cells transfected with HCV RNA and, on the other hand, the absence of lncR 8 induction in cells infected HCV virus at later time points. This may directly lead to the upregulation of ISGs levels in HCV transfected cells, and unaffected level of ISGs mRNA in HCV infected cells. Similar upregulation of ISGs and downregulation of HCV NS3 expression by lncR 8 knockdown were also observed in Huh-7 cells (Figure 8A,G), indicating that lncR 8 is also required for HCV infection in Huh-7 cells and this regulation is independent of *RIG-I*. 

**Figure 14:**
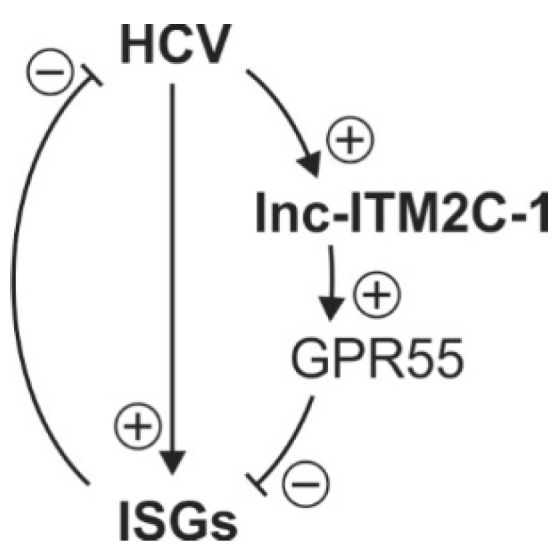
A model depicts the role of lncR 8 and *GPR55* during HCV replication. Indicated is that HCV induces lncR 8/*lnc-ITM2C-1* expression, while lncR 8 favors HCV replication by regulating its neighboring gene *GPR55*, which in turn negatively regulates expression of ISGs.

Considering the different chromosome locations of ISGs and lncR 8, the negative regulation on ISGs by lncR 8 must occur through a *trans*-acting mechanism, which resembles the effect of *EGOT* and *lncRNA-CMPK2*/*NRIR* on HCV [15,29]. Enrichment of lncR 8 in the nucleus (Figure 2B) suggests that the regulation on ISGs could be through regulation of a nuclear event like transcriptional regulation, epigenetic DNA/chromatin modification, or control of pre-mRNA splicing [14,42,47]. To elucidate the possibility that lncR 8 regulates ISGs through its neighboring gene *GPR55*, *GPR55* was inhibited by two different GmRs in Huh-7.5 cells for 48 h. Interestingly, suppression of GPR55 promotes the expression of *ISG15*, *Mix1*, and *IFITM1* (Figure 10). This finding provides a possible link between lncR 8 and ISGs expression, indicating that lncR 8 favors HCV replication by regulating its neighboring gene *GPR55*, which in turn negatively regulates expression of ISGs (Figure 14). Recently, *GPR55* has gained much attention due to its activation by endogenous cannabinoids (EC) and a proinflammatory role in innate immunity [65,66,67]. ECs have been associated with fibrosis progression in HCV-infected patients [68]. On the other hand, elevated levels of ECs were reported in plasma of patients with chronic hepatitis C and indicated potential immunosuppressive and profibrogenic roles [69]. *GPR55* is a third cannabinoid receptor which is novel because it is different from the other two classical receptors, CB_1_ and CB_2_ [67]. High levels of *GPR55* were found in monocyte and natural killer (NK) cells. *GPR55* enhances *IL-12* and *TNF-α* production in monocytes and stimulates signature cytokines as well as cytolytic activity in NK cells [66]. While the detailed function of *GPR55* during HCV replication remains to be determined, the involvement of *GPR55* in the negative regulation of ISGs by lncR 8 indicates a potential anti-inflammatory role of *GPR55* and lncR 8 during early HCV replication. 

Unlike other lncRNAs that can be induced by IFN-α, lncR8 is negatively regulated by IFN-α (Figure 13A) through the JAK-STAT pathway (Figure 13B). We speculated that lncR 8 is normally maintained at low expression level because of IFN-α inhibition. However, we know that irrespective of persistent immune and inflammatory response induced by HCV in vivo, HCV survives in the infected cell. This indicates that HCV develops strategies to bypass the immune response, i.e. release lncR 8 from the control of IFN-α. The innate immune response is insufficient to control viral replication. In the battle of HCV and host cells, increased levels of ISGs should be induced by the immune response against HCV. Most ISGs function by increasing the antiviral response or by inhibiting viral replication. Nevertheless, lncR 8 induced by HCV helps HCV replication by positively regulating its neighboring gene *GPR55*, which in turn negatively regulates ISGs, like *ISG15*, *Mx1*, and *IFITM1*, at early time points. By this mechanism, lncR 8 may contribute to the failure of interferon action and elimination of ongoing HCV infection. Despite of the upregulation of lncR 8 after HCV infection, the *IFITM1* level was still increased at early times (Figure 7F), indicating an involvement of other regulation factors. Though further studies will be required to elucidate the underlying mechanisms, our study benefits a better understanding of lncRNAs in the HCV-host battle.

## Figures and Tables

**Table 1:** Primers for lncRNAs.

Target Gene	Primer Sequences (5′-3′)	Amplicon Size (bp)
LncR 2	F: CTCCCAGAACCTATCGGCAT	130
	R: CACAAAGCCTGCGTTCATTC	
LncR 3	F: AGGATGTGACTGCCAGGTAATG	100
	R: CAGACCCAGCCTAGCACACAG	
LncR 3^3′^	F: GTGACCCAACTAGAGCCAATAGG	135
	R: CTCAAATCAGCTCATGACCATAAG	
LncR 7-1	F: AGGCTACAGGAGGCACTGAGGG	144
	R: GGAGCCATCTGGGAGAATGAAATAC	
LncR 7-2	F: GAGGCTACAGGAGGCACTCTTTG	79
	R: GGAGCCATCTGGGAGAATGAAATAC	
LncR 7^3′^	F: TCGGGTTCTTGATTTGATTCTC	142
	R: TGGACCAAGTATCCTCTAAAAATG	
LncR 8	F: GGTTTTTTGACCTTGGCAATG	102
	R: GTGACCCTTGGTGGCTGTTTAT	
LncR 8^3′^	F: GATTCTGTCTCATCCAATCAAGACT	123
	R: GTTGTGCTGAGGATTCTGGGT	
LncR 10	F: CGGAAATGCCTAATCTGAACTT	80
	R: TAGAGCGGACCCACGAAAC	
LncR 10^3′^	F: CCCCTGATGCTTCATAATGG	111
	R: AGTTCTAACCTAATTTCCCATCAC	

This table lists the sequence of primers for each lncRNA target and the size of amplicons. Two different sets of primers were purchased for lncRs 3, 7, 8, 10. One targets the 5′ end of the sequence, the other the 3′ end. Primers targeting the 3′end of the lncRNA sequences were labeled with 3′. F: Forward, R: Reverse.

**Table 2:** Primers for HCV, reference genes, and ISGs.

Target Gene	Primer Sequences (5′-3′)	Amplicon Size (bp)
*GAPDH*	F: GAGTCAACGGATTTGGTCGT	224
	R: GATCTCGCTCCTGGAAGATG (= RT)	
*U6*	F: CTCGCTTCGGCAGCACA	94
	R: AACGCTTCACGAATTTGCGT	
*U99*	F: CCTCCTTTTCTTGGCGGGGA	138
	R: CGTTTGAGGATAGAACCAGC	
*β-actin*	F: CATGTACGTTGCTATCCAGGC	250
	R: CTCCTTAATGTCACGCACGAT	
Jc1-NS3	RT: GTATGCCACGGCATTCAAG	190
	F: GATATAGGTCGACGGCTCCA	
	R: TTCCTCGGAACAACCATCTC	
*GAS5*	F: CCTGTGAGGTATGGTGCTGG	383
	R: GGTCCAGGCAAGTTGGACTC	
*ITM2C*	F: GTGGTGTGCTGTATGAGGACT	93
	R: CGTAGTTCTCGTCGAGGTAGAT	
*GPR55*	F: GAAAACCCTACAGTTTGCAGTCC	123
	R: GAGGTGGCAGCATAATCGGG	
*CXCL10*	F: GTGGCATTCAAGGAGTACCTC R: TGATGGCCTTCGATTCTGGATT	198
*ISG15*	F: ACTCATCTTTGCCAGTACAGGAG R: CAGCATCTTCACCGTCAGGTC	88
*Mx1*	F: TGCATCGACCTCATTGACTC R: ACCTTGCCTCTCCACTTATC	218
*IFITM1*	F: ACTCCGTGAAGTCTAGGGACA R: AGAGCCGAATACCAGTAACAG	149
*MDA5*	F:TCGAATGGGTATTCCACAGACG	152
	R:GTGGCGACTGTCCTCTGAA	
*IFN-β*	F:GCTTGGATTCCTACAAAGAAGCA	166
	R:ATAGATGGTCAATGCGGCGTC	
*IFN-α*	F: GGAGGTTGTCAGAGCAGA	150
	R: AATGACAGAATTCATGAAAGCGT	
*IL28A*	F: CAGCCTCAGAGTGTTTCTTCT	117
	R: TCCAGTCACGGTCAGCA	

This table lists the sequence of primers and the size of amplicons for targets including HCV NS3 coding region, reference genes and ISGs. F: Forward, R: Reverse, RT: Reverse transcription.

**Table 3:** Characteristics of the lncRNA candidates.

lncRNA	Ref.	Chr	Length(bp)	Gene Symbol	Name
lncR 3	NR_033376.1	6	1753	*lincRNA 222*	*LINC00222*
lncR 7-2	NR_104615.1	5	3451	*LOC100506688*	*Lnc-SLC12A7-4:5*
lncR 8	NR_038238.1	2	1893	*LOC151484*	*Lnc-ITM2C-1*
lncR 10	NR_026974.1	8	3250	*ZNF252P antisense RNA 1*	*ZNF252P-AS1*

Ref.: NCBI Reference Sequence.

**Table 4:** Protein-coding potential of lncRNAs.

Metric	lncR 3	lncR 7-2	lncR 8	lncR 10
CPAT coding probability	1.33%	10.83%	69.31%	80.45%
PhyloCSF score	−67.4569	13.6639	−112.1426	11.7381
PRIDE reprocessing 2.0	0	0	0	0
Lee translation initiation sites	0	0	0	0
Bazzini small ORFs	0	0	0	0

The table lists the results of analysis using different metrics to address the protein-coding potential of candidates from LNCipedia. 5.2 (http://www.lncipedia.org).

## Supplementary Materials

The following are available online at https://www.mdpi.com/1999-4915/11/6/549/s1, Figure S1: Materials related to Figure 3A. Figure S2 (related to Figure 1C, Figure 2A, Figure 4B and Figure 5B). ISGs were upregulated after HCV transfection. (**A**) HCV NS3 level, (**B**) lncR 8, (**C**) lncR 8 neighboring genes ITM2C and GPR55, and (**D**) indicated ISGs in samples treated as described in Figure 1 but without miR-122 were measured 6 days after HCV transfection. qRT-PCR data was normalized to GAPDH. The data are shown as the mean ± SEM of at least three independent experiments. * *p* ≤ 0.05, ** *p* ≤ 0.01, *** *p* ≤ 0.001, and **** *p* ≤ 0.0001.
