# Supplementary material for: Lnc-ITM2C-1 and GPR55 Are Proviral Host Factors for Hepatitis C Virus
Source: Viruses. 2019 Jun 13;11(6):549. doi: 10.3390/v11060549 (PMC6631246; doi:10.3390/v11060549)

Supplementary Figure S1 – related to Fig. 3A

Test for possible crossreactions between GapmeRs:
(https://www.ebi.ac.uk/Tools/services/web_clustalo/toolform.ebi)

```
lncR_7-GmR_2       ----ATAAGTGTCTAGTTAG---------         16
lncR_3-GmR_2       -----GACGATAAGAGGTAAC--------         16
lncR_8-GmR_1       --------GTTACCAGTGAAGCGG-----         16
Random_GmR_        ----AACAC-----GTCTATACGC-----         15
lncR_3-GmR_1       ----------GCGTGATTAAATGGAT---         16
lncR_7-GmR_1       -------------TGATTAACAGAACGGA         16
lncR_8-GmR_2       -------TCGGATTGGTCACATG------         16
lncR_10-GmR_1      GTTAATCTGATCTTGC-------------         16
lncR_10-GmR_2      -----TCTGAGCTTGATCACT--------         16
```

Conclusion:
All used GapmeRs do not show any crossreactions among each other.

GapmeR specificities
GapmeR sequences were matched against Human genomic plus transcript (Human G-T) sequences using NBLAST (https://blast.ncbi.nlm.nih.gov/Blast.cgi?PROGRAM=blastn&PAGE_TYPE=BlastSearch&LINK_LOC=blasthome)using default parameters but without any filtering of repeat sequences. For each GapmeR, the best 5 matches to human transcripts (except the genuine target) are shown.

Conclusions (for details please see below):
The Randomized control GapmeR does not bind specifically to any target in the human transcriptome. Virtually all GapmeRs specific for lncRNAs used in this study are very specific for their genuine targets. Only lncR 10-GmR 1 can bind to only one other target RNA with a temperature difference of only at least 1 °C (value calculated for DNA), to the next target with at least 5 °C difference.

Random_GmR; Query: AACACGTCTATACGC (15 nts)
5 best matches (binding to RNA splice variants removed from output):

Homo sapiens zinc finger CCHC-type containing 23 (ZCCHC23), mRNA, Sequence ID: NM_001039778.2
```
Query  1    AACACGTCTATAC  13
            |||||||||||||
Sbjct  973  AACACGTCTATAC  961
```

PREDICTED: Homo sapiens uncharacterized LOC107984587 (LOC107984587), ncRNA
Sequence ID: XR_001749928.1
```
Query  1    AACACGTCTATAC  13
            |||||||||||||
Sbjct  578  AACACGTCTATAC  590
```

Homo sapiens long intergenic non-protein coding RNA 645 (LINC00645), long non-coding RNA
Sequence ID: NR_039992.2
```
Query  1     AACACGTCTATAC  13
             |||||||||||||
Sbjct  1476  AACACGTCTATAC  1464
```

PREDICTED: Homo sapiens DNA polymerase eta (POLH), transcript variant X2, mRNA
Sequence ID: XM_024446466.1
```
Query  1     AACACGTCTATA  12
             ||||||||||||
Sbjct  3057  AACACGTCTATA  3046
```

Homo sapiens chromosome 2, GRCh38.p12 Primary Assembly
Sequence ID: NC_000002.12
```
Query  1          AACACGTCTATACG  14
                  ||||||||||||||
Sbjct  112349170  AACACGTCTATACG  112349183
```

Conclusion: Random_GmR (15 nts) has at least 2 mismatches to any human transcript. For standard DNA oligonucleotides this would correspond to a drop in T_M by 11 °C compared to full hybridization.

lncR 3-GmR 1; Query: GCGTGATTAAATGGAT (16 nts)
5 best matches (except genuine target, binding to RNA splice variants removed from output):

PREDICTED: Homo sapiens EYA transcriptional coactivator and phosphatase 4 (EYA4), transcript variant X1, mRNA, Sequence ID: XM_017010368.2
```
Query  3   GTGATTAAATGGAT  16
           ||||||||||||||
Sbjct  60  GTGATTAAATGGAT  7
```

PREDICTED: Homo sapiens macrophage scavenger receptor 1 (MSR1), transcript variant X1, mRNA, Sequence ID: XM_024447160.1
```
Query  4     TGATTAAATGGAT  16
             |||||||||||||
Sbjct  1554  TGATTAAATGGAT  1542
```

PREDICTED: Homo sapiens transmembrane protein 181 (TMEM181), transcript variant X5, mRNA, Sequence ID: XM_005267074.4
```
Query  4     TGATTAAATGGAT  16
             |||||||||||||
Sbjct  2295  TGATTAAATGGAT  2307
```

PREDICTED: Homo sapiens zinc finger FYVE-type containing 16 (ZFYVE16), transcript variant X18, mRNA, Sequence ID: XM_024446273.1
```
Query  4     TGATTAAATGGAT  16
             |||||||||||||
Sbjct  8457  TGATTAAATGGAT  8469
```

PREDICTED: Homo sapiens SMAD family member 5 (SMAD5), transcript variant X3, mRNA, Sequence ID: XM_024446047.1
```
Query  4     TGATTAAATGGAT  16
             |||||||||||||
Sbjct  4433  TGATTAAATGGAT  4421
```

Conclusion: lncR 3-GmR 1 (16 nts) has at least 2 mismatches to any other human transcript. For standard DNA oligonucleotides this would correspond to a drop in T_M by 10 °C compared to full hybridization.

lncR 3-GmR 2; Query: GACGATAAGAGGTAAC (16 nts)
5 best matches (except genuine target, binding to RNA splice variants removed from output):

PREDICTED: Homo sapiens PRELI domain containing 2 (PRELID2), transcript variant X2, mRNA, Sequence ID: XM_017009127.1
```
Query  4    GATAAGAGGTAAC  16
            |||||||||||||
Sbjct  982  GATAAGAGGTAAC  994
```

PREDICTED: Homo sapiens chromosome 8 open reading frame 34 (C8orf34), transcript variant X3, misc_RNA, Sequence ID: XR_928756.3
```
Query  4     GATAAGAGGTAA  15
             ||||||||||||
Sbjct  6998  GATAAGAGGTAA  7009
```

PREDICTED: Homo sapiens inner mitochondrial membrane peptidase subunit 2 (IMMP2L), transcript variant X13, mRNA, Sequence ID: XM_024446959.1
```
Query  5     ATAAGAGGTAAC  16
             ||||||||||||
Sbjct  2239  ATAAGAGGTAAC  2250
```

PREDICTED: Homo sapiens Cbl proto-oncogene like 1 (CBLL1), transcript variant X7, mRNA, Sequence ID: XM_011516580.3
```
Query  3     CGATAAGAGGTA  14
             ||||||||||||
Sbjct  1974  CGATAAGAGGTA  1963
```

PREDICTED: Homo sapiens parkin coregulated (PACRG), transcript variant X6, mRNA, Sequence ID: XM_011535461.3
```
Query  5     ATAAGAGGTAAC  16
             ||||||||||||
Sbjct  2089  ATAAGAGGTAAC  2100
```

Conclusion: lncR 3-GmR 2 (16 nts) has at least 3 mismatches to any other human transcript. For standard DNA oligonucleotides this would correspond to a drop in T_M by 11 °C compared to full hybridization.

lncR 7-GmR 1; Query: TGATTAACAGAACGGA (16 nts)
5 best matches (except genuine target, binding to RNA splice variants removed from output):

PREDICTED: Homo sapiens MCF.2 cell line derived transforming sequence (MCF2), transcript variant X6, mRNA, Sequence ID: XM_017029532.2
```
Query  3     ATTAACAGAACGG  15
             |||||||||||||
Sbjct  2871  ATTAACAGAACGG  2883
```

PREDICTED: Homo sapiens proline rich 26 (PRR26), transcript variant X4, mRNA, Sequence ID: XM_024448024.1
```
Query  1    TGATTAACAGAAC  13
            |||||||||||||
Sbjct  854  TGATTAACAGAAC  866
```

PREDICTED: Homo sapiens uncharacterized LOC285500 (LOC285500), transcript variant X3, mRNA, Sequence ID: XM_011532460.2
```
Query  1     TGATTAACAGAAC  13
             |||||||||||||
Sbjct  1435  TGATTAACAGAAC  1423
```

PREDICTED: Homo sapiens UTP18, small subunit processome component (UTP18), transcript variant X2, mRNA, Sequence ID: XM_011524870.2
```
Query  4     TTAACAGAACGGA  16
             |||||||||||||
Sbjct  1542  TTAACAGAACGGA  1554
```

PREDICTED: Homo sapiens mal, T cell differentiation protein like (MALL), transcript variant X1, mRNA, Sequence ID: XM_011511809.1
```
Query  2    GATTAACAGAACG  14
            |||||||||||||
Sbjct  602  GATTAACAGAACG  614
```

Conclusion: lncR 7-GmR 1 (16 nts) has at least 3 mismatches to any other human transcript. For standard DNA oligonucleotides this would correspond to a drop in T_M by 9 °C compared to full hybridization.

lncR 7-GmR 2; Query: ATAAGTGTCTAGTTAG (16 nts)
5 best matches (except genuine target, binding to RNA splice variants removed from output):

PREDICTED: Homo sapiens myotubularin related protein 10 (MTMR10), transcript variant X4, mRNA, Sequence ID: XM_005254508.3
```
Query  3     AAGTGTCTAGTTAG  16
             ||||||||||||||
Sbjct  3699  AAGTGTCTAGTTAG  3712
```

Homo sapiens PCNA clamp associated factor (PCLAF), transcript variant 3, non-coding RNA, Sequence ID: NR_109934.1
```
Query  2    TAAGTGTCTAGTT  14
            |||||||||||||
Sbjct  768  TAAGTGTCTAGTT  780
```

Homo sapiens proline rich 9 (PRR9), mRNA, Sequence ID: NM_001195571.1
```
Query  1    ATAAGTGTCTAGT  13
            |||||||||||||
Sbjct  184  ATAAGTGTCTAGT  196
```

PREDICTED: Homo sapiens chromosome X open reading frame 38 (CXorf38), transcript variant X5, mRNA, Sequence ID: XM_006724527.4
```
Query  5     GTGTCTAGTTAG  16
             ||||||||||||
Sbjct  3143  GTGTCTAGTTAG  3154
```

PREDICTED: Homo sapiens branched chain keto acid dehydrogenase E1 subunit beta (BCKDHB), transcript variant X6, mRNA, Sequence ID: XM_011536023.3
```
Query  3     AAGTGTCTAGTT  14
             ||||||||||||
Sbjct  1536  AAGTGTCTAGTT  1525
```

Conclusion: lncR 7-GmR 2 (16 nts) has at least 2 mismatches to any other human transcript. For standard DNA oligonucleotides this would correspond to a drop in T_M by 1 °C compared to full hybridization.

lncR 8-GmR 1; Query: GTTACCAGTGAAGCGG (16 nts)
5 best matches (except genuine target, binding to RNA splice variants removed from output):

PREDICTED: Homo sapiens schlafen family member 12 (SLFN12), transcript variant X6, mRNA, Sequence ID: XM_017024811.2

```
Query  1    GTTACCAGTGAAG  13
            |||||||||||||
Sbjct  452  GTTACCAGTGAAG  440
```

PREDICTED: Homo sapiens citrate lyase beta like (CLYBL), transcript variant X10, mRNA, Sequence ID: XM_024449330.1

```
Query  2     TTACCAGTGAAGC  14
             |||||||||||||
Sbjct  1682  TTACCAGTGAAGC  1670
```

PREDICTED: Homo sapiens COP1, E3 ubiquitin ligase (COP1), transcript variant X25, mRNA, Sequence ID: XM_017002080.2

```
Query  1     GTTACCAGTGAAG  13
             |||||||||||||
Sbjct  5564  GTTACCAGTGAAG  5576
```

PREDICTED: Homo sapiens uncharacterized LOC105375318 (LOC105375318), transcript variant X1, ncRNA, Sequence ID: XR_001745236.1

```
Query  2    TTACCAGTGAAGC  14
            |||||||||||||
Sbjct  572  TTACCAGTGAAGC  584
```

PREDICTED: Homo sapiens uncharacterized LOC105375101 (LOC105375101), ncRNA
Sequence ID: XR_001744179.1

```
Query  2    TTACCAGTGAAGC  14
            |||||||||||||
Sbjct  866  TTACCAGTGAAGC  878
```

Conclusion: lncR 8-GmR 1 (16 nts) has at least 3 mismatches to any other human transcript. For standard DNA oligonucleotides this would correspond to a drop in T_M by 12 °C compared to full hybridization.

lncR 8-GmR 2; Query: TCGGATTGGTCACATG (16 nts)
5 best matches (except genuine target, binding to RNA splice variants removed from output):

PREDICTED: Homo sapiens LIF receptor alpha (LIFR), transcript variant X4, mRNA, Sequence ID: XM_011514042.3

```
Query  4     GATTGGTCACATG  16
             |||||||||||||
Sbjct  6201  GATTGGTCACATG  6213
```

PREDICTED: Homo sapiens regulator of G protein signaling 9 (RGS9), transcript variant X1, mRNA, Sequence ID: XM_011525426.3

```
Query  1     TCGGATTGGTCAC  13
             |||||||||||||
Sbjct  1080  TCGGATTGGTCAC  1068
```

Homo sapiens long intergenic non-protein coding RNA 1550 (LINC01550), transcript variant 2, long non-coding RNA, Sequence ID: NR_152746.1

```
Query  3     GGATTGGTCACAT  15
             |||||||||||||
Sbjct  1862  GGATTGGTCACAT  1850
```

Homo sapiens dispatched RND transporter family member 1 (DISP1), transcript variant 2, mRNA, Sequence ID: NM_001350630.1

```
Query  4    GATTGGTCACATG  16
            |||||||||||||
Sbjct  891  GATTGGTCACATG  903
```

Homo sapiens glutathione peroxidase 3 (GPX3), transcript variant 1, mRNA
Sequence ID: NM_002084.4

```
Query  2    CGGATTGGTCACA  14
            |||||||||||||
Sbjct  127  CGGATTGGTCACA  139
```

Conclusion: lncR 8-GmR 2 (16 nts) has at least 3 mismatches to any other human transcript. For standard DNA oligonucleotides this would correspond to a drop in T_M by 5 °C compared to full hybridization.

lncR 10-GmR 1; Query: GTTAATCTGATCTTGC (16 nts)
5 best matches (except genuine target, binding to RNA splice variants removed from output):

PREDICTED: Homo sapiens solute carrier family 4 member 4 (SLC4A4), transcript variant X6, mRNA, Sequence ID: XM_024454272.1
```
Query  2     TTAATCTGATCTTGC  16
             |||||||||||||||
Sbjct  4333  TTAATCTGATCTTGC  4319
```

PREDICTED: Homo sapiens tRNA-yW synthesizing protein 5 (TYW5), transcript variant X2, misc_RNA, Sequence ID: XR_001738610.2
```
Query  2     TTAATCTGATCTTG  15
             ||||||||||||||
Sbjct  1979  TTAATCTGATCTTG  1992
```

PREDICTED: Homo sapiens embigin (EMB), transcript variant X1, mRNA, Sequence ID: XM_011543146.2
```
Query  2     TTAATCTGATCTTG  15
             ||||||||||||||
Sbjct  3760  TTAATCTGATCTTG  3773
```

PREDICTED: Homo sapiens roundabout guidance receptor 2 (ROBO2), transcript variant X26, mRNA, Sequence ID: XM_017007006.1
```
Query  3     TAATCTGATCTTGC  16
             ||||||||||||||
Sbjct  7680  TAATCTGATCTTGC  7693
```

PREDICTED: Homo sapiens ATPase family, AAA domain containing 2 (ATAD2), transcript variant X4, misc_RNA, Sequence ID: XR_928326.3
```
Query  4     AATCTGATCTTGC  16
             |||||||||||||
Sbjct  1731  AATCTGATCTTGC  1719
```

Conclusion: lncR 10-GmR 1 (16 nts) has at least 1 mismatch to any other human transcript. For standard DNA oligonucleotides this would correspond to a drop in T_M by 3 °C compared to full hybridization. Hybridization to the next target (2 mismatches) would correspond to a drop in T_M by 9 °C.

lncR 10-GmR 2; Query: TCTGAGCTTGATCACT (16 nts)
5 best matches (except genuine target, binding to RNA splice variants removed from output):

PREDICTED: Homo sapiens solute carrier family 35 member B4 (SLC35B4), transcript variant X2, misc_RNA, Sequence ID: XR_001744887.2
```
Query  3     TGAGCTTGATCACT  16
             ||||||||||||||
Sbjct  1725  TGAGCTTGATCACT  1738
```

PREDICTED: Homo sapiens ring finger protein 14 (RNF14), transcript variant X6, mRNA, Sequence ID: XM_005268541.4
```
Query  2    CTGAGCTTGATCA  14
            |||||||||||||
Sbjct  619  CTGAGCTTGATCA  607
```

PREDICTED: Homo sapiens uncharacterized LOC107986317 (LOC107986317), ncRNA, Sequence ID: XR_002959804.1
```
Query  4     GAGCTTGATCACT  16
             |||||||||||||
Sbjct  1859  GAGCTTGATCACT  1847
```

PREDICTED: Homo sapiens OPA1, mitochondrial dynamin like GTPase (OPA1), transcript variant X2, misc_RNA, Sequence ID: XR_001740159.2
```
Query  2     CTGAGCTTGATCA  14
             |||||||||||||
Sbjct  6007  CTGAGCTTGATCA  6019
```

PREDICTED: Homo sapiens neuron navigator 3 (NAV3), transcript variant X14, mRNA, Sequence ID: XM_017020173.2
```
Query  1     TCTGAGCTTGATC  13
             |||||||||||||
Sbjct  1388  TCTGAGCTTGATC  1376
```

Conclusion: lncR 10-GmR 2 (16 nts) has at least 2 mismatches to any other human transcript. For standard DNA oligonucleotides this would correspond to a drop in T_M by 5 °C compared to full hybridization.

Figure S2 (related to Fig. 1C, 2A, 4B and 5B**).** ISGs were upregulated after HCV transfection. (**A**) HCV NS3 level, (**B**) lncR 8, (**C**) lncR 8 neighboring genes ITM2C and GPR55, and (**D**) indicated ISGs in samples treated as described in Figure 1 but without miR-122 were measured 6 days after HCV transfection**.** qRT-PCR data was normalized to GAPDH. The data are shown as the mean ± SEM of at least three independent experiments. * $p \leq 0.05$, ** $p \leq 0.01$, *** $p \leq 0.001$, and **** $p \leq 0.0001$.

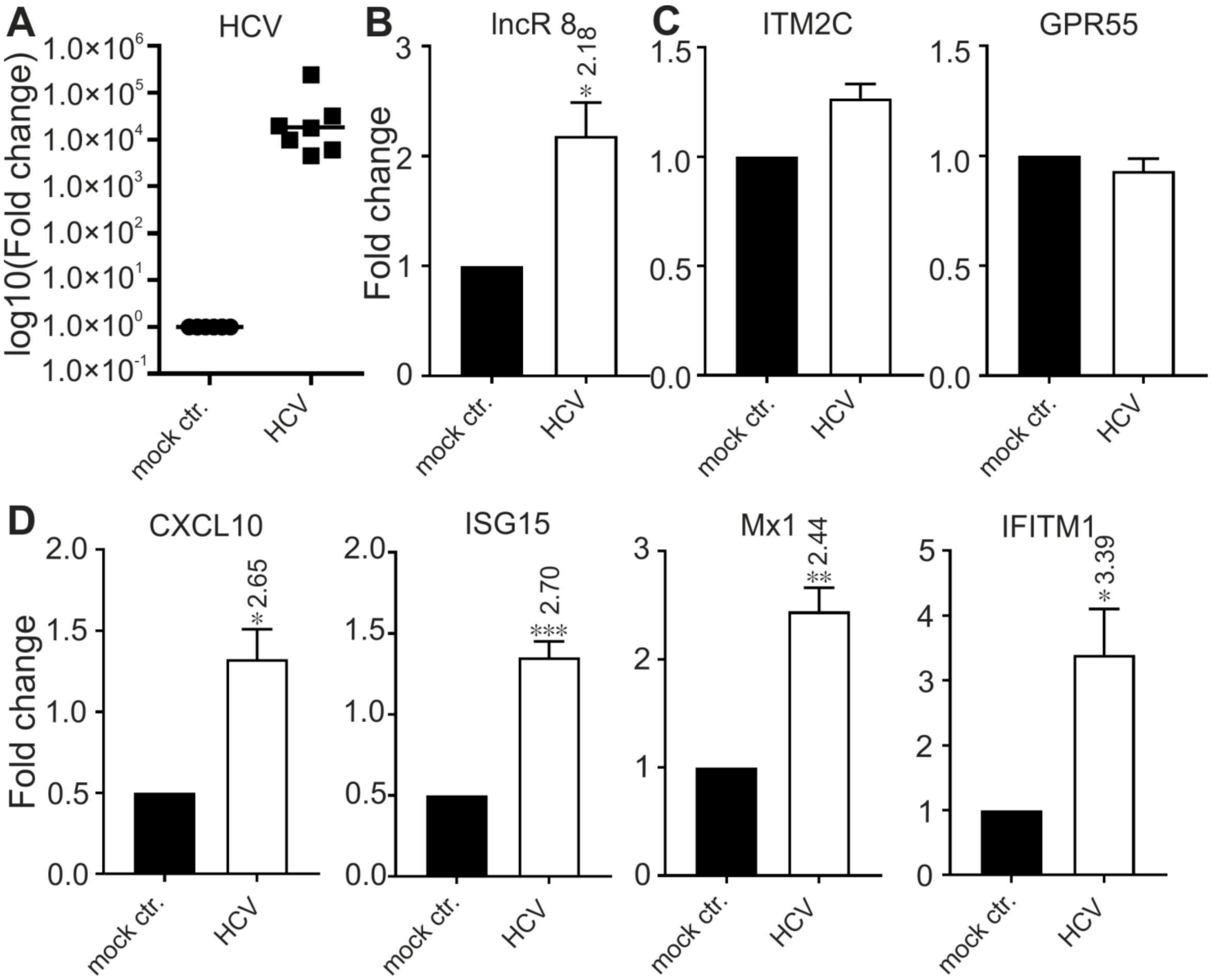

Supplement: Supplementary file 1 [file viruses-11-00549-s001.pdf]
